# Recent Developments in Novel TPMS Lattice Materials: Design Optimization, Performance Control, and Applications in Biomimetic Scaffolds

**DOI:** 10.3390/ma18225209

**Published:** 2025-11-17

**Authors:** Syed Zahid Ahmad, Muhammad Hassan Masood, Muhammad Umar Khattab, Syed Sulman Ahmad, Syed Asad Ali Zaidi, Sohaib Z. Khan

**Affiliations:** 1 School of Mechanical, Aerospace and Manufacturing Engineering, University of Connecticut, Storrs, CT 06269, USA; uxm24002@uconn.edu; 2 Department of Mechanical Engineering, National University of Sciences and Technology, Islamabad 44000, Pakistan; 3 Department of Mechanical Engineering, University of Washington, Room G33, 3900 E Stevens Way NE, Seattle, WA 98195, USA; syedsulm@uw.edu; 4 Department of Mechanical Engineering, Islamic University of Madinah, Madinah 42351, Saudi Arabia; sali@iu.edu.sa

**Keywords:** TPMS scaffolds, biomimetic scaffolds, design methods, TPMS applications, IPCs, heat sinks, manufacturing, topology optimization, multi-scale optimization

## Abstract

Triply Periodic Minimal Surfaces (TPMSs) are mathematically defined surfaces that exhibit periodicity in three dimensions while maintaining a minimal surface property. TPMS-based lattices have gained significant attention in recent years, fueled by advancements in Additive Manufacturing (AM). These structures exhibit exceptional mechanical, thermal, and mass transfer properties, positioning them as a promising class of next-generation materials. However, fully leveraging their potential requires a comprehensive understanding of their design, properties, optimization, and applications. Given the hierarchical nature of TPMSs, achieving optimal performance requires multiscale optimization at the macro- and micro-levels. Addressing these complexities requires advanced computational methods to balance structural integrity and functional performance. In this narrative review, design strategies like functional grading and hybridization to create optimized TPMS-based lattices are summarized. Herein, the performance of such lattices in the mechanical, thermal, and mass transfer domains is focused upon. The role of topology optimization (TO) in the creation of architectured materials for specific application is discussed along with the emerging integration of machine learning. Furthermore, multidisciplinary applications of TPMS structures are examined, particularly in heat sinks, interpenetrating phase composites (IPCs), and biomimetic scaffolds, with their potential to enhance heat dissipation, structural resistance, and biomimicry of biological scaffolds. In addition, various additive manufacturing technologies for fabricating TPMS structures are reviewed, emphasizing how additive manufacturing allows high reproducibility construction of their complex geometry in a precise manner. Further unexplored areas of research are also discussed.

## 1. Introduction

The majority of the materials utilized in the industry are considered to be solid and have few porosities. However, there are many porous structures that occur naturally with excellent characteristics, e.g., coral bones, woods, and honeycombs [1]. The global shift towards lighter and stronger materials has created a critical demand for the development of advanced artificial cellular structures. These structures can be classified into foams, honey combs, and lattices. Among all three, the lattices provide the best properties to be used in different applications such as aviation, automotive, and other industrial fields. The design of lattice structures was restricted because of manufacturing limitations, and traditional methods cannot contribute to the manufacturing of such structures. Due to the advancement in AM, new avenues for the manufacturing of such porous structures are being explored [2]. In recent years, architectured materials have gained significant attention due to their mathematically controlled structures and superior mechanical, heat transfer, and mass transfer properties. However, there are still questions regarding the design of lattices, their optimization, and their broad applications.

TPMSs are given by trigonometric equations, characterized by a mean curvature of zero and a high surface area-to-volume ratio [3]. They are geometrically intricate structures which offer wide ranging applications in materials science, architecture, and bioengineering. These structures are lightweight and can replace materials where weight is of great concern, but a trade-off between performance and structural integrity always exists. Their equations offer great flexibility to adjust their microstructure, such as adjusting the thickness of walls and varying the unit cell size. If the change is gradual, they are known as functionally graded lattice structures. TPMS lattices have superior performance behaviors and have strong potential to substitute or, in appropriately optimized scenarios, substitute traditional materials in structural design applications, heat transfer applications, and mass transfer applications. Their application in load-bearing or high-performance applications, however, is typically limited by specific failure modes, including local buckling due to compressive loads, notch sensitivities due to sharp curvature changes, and processing-induced flaws like surface roughness, porosity variation, and partially melted particles characteristic to AM processes. Such flaws have the potential to induce premature failure or decrease fatigue life, thereby necessitating careful design verification and post-processing. However, to fully exploit their advantages, it is essential to optimize these structures for specific applications. This is where structural optimization becomes essential, allowing the design of TPMS-based lattice structures with enhanced performance goals, such as improved weight-to-strength ratio, better heat dissipation, etc. Among the various methods of structural optimization, topology optimization remains the most widely used and effective approach.

TO employs different methods and algorithms to update the analysis with design changes. The most prevalent methods are density-based methods, which divide the design domain into finite numbers of elements, in which each element is assigned a density variable that ranges from 0 (empty) to 1 (filled). Level-set methods define the structural boundary using the zero-level-set of a function. A recently developed optimization method, known as the feature mapping method as given by [4], uses geometric feature parameterization, which is then mapped onto a fixed grid for analysis.

Although TO techniques can produce good results, there exist several challenges in them, such as connectivity problems and a high computational cost. Finite element analysis (FEA) contributes primarily to the high computational cost [5]. The work is being done on various methods to minimize the computational cost of TO, with artificial intelligence emerging as the leading approach. AI encompasses technologies that enable machines to replicate human behavior. Within AI, machine learning focuses on identifying meaningful patterns in data using statistical techniques. A further subset, deep learning, is inspired by the structure of the human brain and enhances learning capabilities by training hierarchical neural networks with multiple layers, allowing the system to learn directly from data [6].

TPMS lattices are being utilized more and more in engineering and biomedical applications. In thermal management, TPMS-based heat sinks have improved heat dissipation over the traditional flat-plate or pin-fin geometries due to their porosity and continuity. It has been established through experiments that gyroid-based TPMS heat sinks, through TO, have better thermal conductivity and minimum maximum temperatures under the same design constraints [7,8]. TPMS structures, with these properties, are appropriate for next-generation thermal systems in the electronics, automotive, and aerospace sectors, where compactness and efficiency are critical [9,10].

In materials science, TPMS structures are being utilized to enhance the performance of IPCs by maximizing their mechanical properties, such as energy absorption and compressive strength. AM allows precise production of TPMS-strengthened IPCs, where aluminum–alumina composites show up to 6.79% improvement in energy absorption for gyroid and diamond structures compared to primitive TPMSs [11]. TPMS lattices are equally important in developing biomedical scaffolds, especially for orthopedic use. Their porosity and mechanical properties can be tuned, allowing for effective nutrient delivery and adhesion of cells, capable of mimicking the behavior of native bone tissue. Graded TPMS scaffolds improve better stress distribution and mechanical compatibility with bone structures, thus being potential candidates for tailored implants [12,13].

It was only possible to realize complex TPMS geometries with the help of AM technologies that have transcended traditional methods such as machining and casting [14]. By fabricating materials layer by layer from computer-aided design models, AM provides fine control over pore geometry, unit cell size, and morphology of the surface essential parameters to attain the desired mechanical, thermal, and biological performance. Stereolithography (SLA) and Digital Light Processing (DLP) processes have gained widespread popularity in fabricating polymeric and ceramic TPMS due to high resolution and smooth surfaces that afford bioceramic and hydrogel scaffolds with controllable porosity and bone-mimicking mechanical behavior [15,16]. In the case of metallic TPMS, Selective Laser Melting (SLM) and Electron Beam Melting (EBM) have shown high structural fidelity and mechanical properties close to trabecular bone while facilitating defect-free, load-carrying lattice construction [17,18]. Selective Laser Sintering (SLS) of polymer TPMS, Fused Deposition Modeling (FDM) of inexpensive thermoplastic scaffolds, and Robocasting for bioactive ceramics enables greater versatility in manufacturing TPMS structures [19]. SLS has also produced gyroid-based thermal insulation materials with low thermal properties [20,21]. In short, these AM approaches have eliminated the disconnect between computational TPMS design and real-world fabrication, and it is possible to realize light-weight, high-performance structures with customized designs suitable for various engineering and biomedical applications.

In this review paper, we will analyze the design methods of lattice structures that are based on TPMSs, as well as their performances, optimization, manufacturing techniques, and applications.

## 2. Classification of Lattice Structures

**Figure 1:**
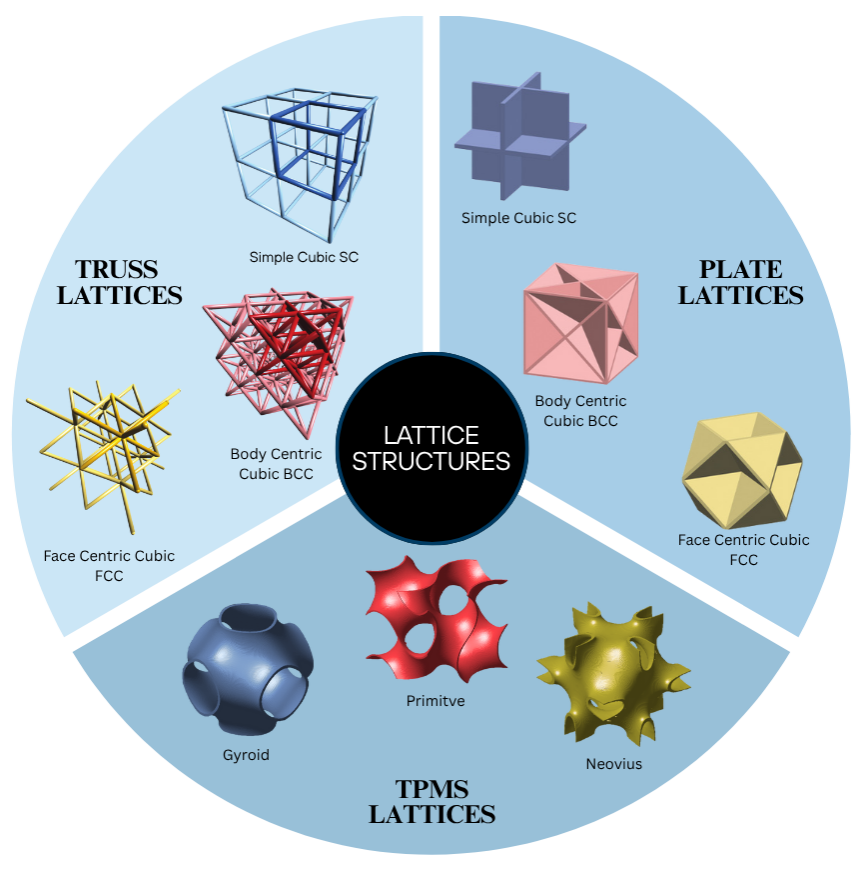
Three main classes of lattice structures.

Lattices are a special type of cellular structures. They are defined as periodic arrangements of unit cells in three dimensions. They can be classified into periodic and non-periodic or stochastic and can be made up of beams/struts, plates, and surfaces. Based on their appearance, we have divided the lattice structures into three main types: truss, plate, and TPMS in Figure 1.

### 2.1. Truss Lattices

Truss lattices consist of struts that are connected at nodes; the number of struts and nodes define their mechanical properties. The most common type are elementary cubic trusses, which consist of Simple cubic (SC), Body-centric Cubic, and Face, centric cubic. Other variants include non-cubic trusses such as Simple Orthorhombic (SO), Simple Tetragonal (ST), and Rhombohedral (R) configurations. These geometries can also be integrated to form compound truss structures with enhanced mechanical characteristics [22]. Finite element software can also be utilized to generate optimized truss lattice configurations through TO techniques, enabling the design of lightweight structures tailored for mechanical performance [23].

### 2.2. Plate Lattices

Plate lattices are a relatively new type of architected materials. They are created by placing thin plates across planes of cubic lattices. These structure achieve near-optimal isotropic stiffness and yield strength, outperforming truss lattices of the same density [24]. Ref. [25] identifies a new class of a hybrid plate lattice cubic and octet foam that achieves the Hashin–Shtrikman upper bounds for isotropic elastic stiffness. Plate lattices with closed structures are difficult to manufacture using any 3D printing technique, so ref. [26] introduced a new type of semi-plate lattices that overcomes fabrication limitations. Using multi-jet 3D printing and wax removal methods, these lattices can be manufactured at centimeter scale with superior stiffness, strength, and energy absorption compared to truss lattices.

### 2.3. TPMS Lattices

TPMS lattices are defined by TPMS surfaces. TPMS-based lattices show great advantages in the structure, heat, and mass transfer applications. The Schwarz P surface, proposed by Hermann Amandus Schwarz in 1865, has cubic symmetry and serves as a foundational structure. Similarly, Schwarz introduced the Schwarz D (Diamond) surface in 1866, characterized by its diamond-like topology. Another well-known surface, the Schwarz Gyroid (G), was proposed by Alan Schoen in 1970 [3]. This surface has become widely used in modern material design. Today, more than 100 TPMSs have been discovered. Of all these surfaces, Gyroid (G), Diamond (D), and Schwarz Primitive (P) are the most commonly used lattice structures.

**Figure 2:**
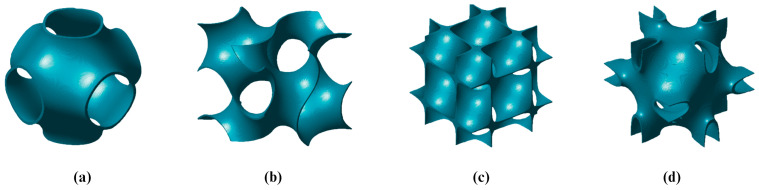
TPMSs. (**a**) Primitive. (**b**) Gyroid. (**c**) Diamond. (**d**) Neovius.

The topology of TPMSs is characterized by zero mean curvature, indicating that the sum of the principal curvatures at any given point on the surface is equal to zero. Having a smooth curvature reduces the stress concentration commonly found in other structures. Minimal surfaces can be repeated with different periodicities and relative densities to obtain desired properties. Some of the TPMSs are shown in Figure 2.

Let $W=\frac{2\pi }{L}$ denote the spatial frequency (wavenumber) corresponding to the unit cell length *L*. Then, the implicit equations of the TPMS are given by

(1) Primitive (P) Surface:cos(WX)+cos(WY)+cos(WZ)=c

(2) Gyroid (G) Surface:sin(WX)cos(WY)+sin(WZ)cos(WX)+sin(WY)cos(WZ)=c

(3) Diamond (D) Surface:cos(WX)cos(WY)cos(WZ)−sin(WX)sin(WY)sin(WZ)=c

(4) NeoviusSurface:cos(WX)+cos(WY)+cos(WZ)+4cos(WX)cos(WY)cos(WZ)=c

where X,Y,Z are Cartesian coordinates in three-dimensional space, *L* is the unit cell size, $W=\frac{2\pi }{L}$ is the spatial frequency or wavenumber, and *c* is the level-set constant determining the relative density.

**Figure 3:**
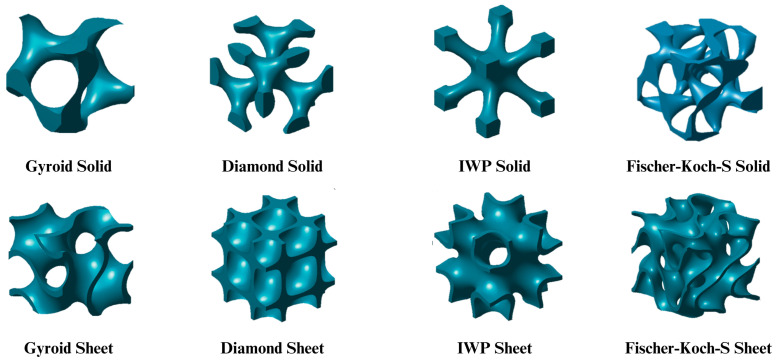
Skeleton-based (solid) vs. sheet-based TPMS Structures.

It should be noted that TPMS-based lattices can be divided into skeleton and sheet-based. In skeleton-based (or solid-based) TPMS structures, the minimal surface divides the space into two distinct regions: one is filled with material and the other remains empty. The sheet-based TPMS is formed by applying offset along the normal vector of the surface. Examples of both are shown in Figure 3. These two types of TPMS structures exhibit distinct properties. Experimental studies by [27] comparing the mechanical properties of sheet-based and skeleton-based TPMS structures demonstrated that sheet-based Diamond TPMSs closely matched the mechanical properties of natural bone. The study applied uniaxial compression along the printing axis. Sheet-Diamond structures with lower porosity showed significantly higher yield strength and stiffness, attributed to reduced buckling and improved load distribution. Similarly. [28] conducted mechanical tests by applying uniaxial loading, comparing scaffolds of both in Diamond, Gyroid, and I-WP cell architectures, concluding that being sheet-based generally demonstrated superior mechanical properties. The study by [29] reveals that sheet-like TPMS scaffolds outperform skeletal variants due to their higher surface area-to-volume ratios, smoother curvature, and interconnected porosities, making them ideal for bone and cartilage regeneration.

### 2.4. Performance of TPMS Lattices

The performance of uniform TPMS structures (i.e., structure that consist of a single type of TPMS) has emerged as a key research area in recent studies. Depending on the application, it is necessary to study mechanical, thermal, optical, or multiphysics coupling performances. Different performance control strategies are being developed which aim to develop TPMS porous structures suitable for engineering applications.

#### 2.4.1. Mechanical Performance

**Figure 4:**
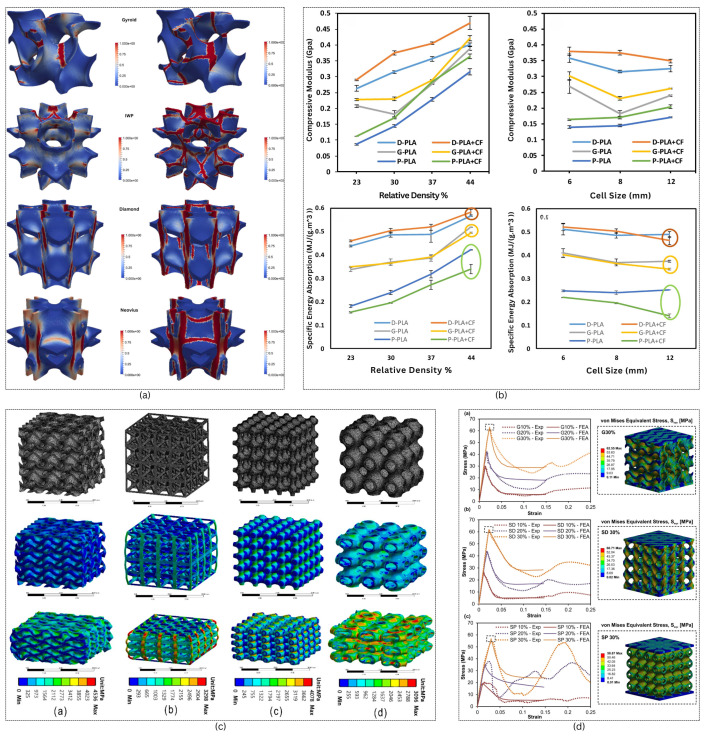
(**a**) Fracture progression in the Gyroid, IWP, Diamond, and Neovius TPMS unit cell for 0.75% and 1.5% of strain. Reproduced from [36], licensed under CC BY 4.0. (**b**) Compressive strength and specific energy absorption of Diamond, Gyroid, and Primitive, fabricated using fused deposition modeling (FDM) with polylactic acid (PLA) and carbon fiber-reinforced PLA (PLA + CF). Reproduced from [38], licensed under CC BY 4.0. (**c**) Finite element analysis illustrating meshing and stress distribution under applied strain for Gyroid, BCC, Primitive, and Truss lattice structures. Reproduced from [33], licensed under CC BY 4.0. (**d**) Mechanical behavior of PLA-based TPMS structures under quasi-static compression. Reproduced from [39], licensed under CC BY 4.0.

TPMS structures have demonstrated notable advantages over conventional designs in multiple performance evaluations, particularly in mechanical properties. Research by [30] revealed that TPMS architectures, characterized by their lack of nodal points, show enhanced fatigue resistance when compared to octahedron unit cell configurations. In studies of viscoelastic performance, ref. [31] established that sheet-IWP TPMS variants exhibit superior behavior under uniaxial loading conditions relative to traditional lattice-frame materials. Ref. [32] compared various lattices and showed that sheet-based Gyroid provides optimal stability and the most extensive range of mechanical properties among various designs, including cubic and octet-strut-based structures. Further reinforcing these findings, ref. [33] documented that sheet-based Gyroid TPMS structures surpass both BCC and truss lattice designs across multiple metrics including stiffness, yield stress, and toughness, as illustrated in Figure 4c. The material used is 316L stainless steel, fabricated via Selective Laser Melting (SLM). In the compression tests, boundary conditions include a fixed bottom plate and a uniformly loaded top plate, simulating uniaxial compression. The mesh consists of tetrahedral Solid187 elements with an average size of 0.5 mm. Finite element simulations were conducted using ANSYS Workbench 2020R2, utilizing isotropic hardening and full-size models to capture deformation and stress distribution. Ref. [34] shows that both yield strength and Young’s modulus decrease as the unit cell size increases, while compressive strength rises with higher volume fractions in a 3D-printed gyroid structure. Similarly a Neovius structure manufactured by fused deposition modeling showed the same behavior under compressive loading [35]. The study [36] demonstrates that TPMS-based open-cell foams, specifically Primitive, Gyroid, IWP, Diamond, and Neovius structures, exhibit superior fracture resistance and compressive mechanical properties compared to conventional aluminum foams, as shown in Figure 4a. The study investigates the fracture behavior of TPMS structures using aluminum alloy (Al–Si10–Mg) with material properties: a Young’s modulus of 82 GPa, Poisson’s ratio of 0.33, and fracture toughness of approximately $10^{-4}$ J/mm^2^. The unit cells of TPMS (Primitive, Gyroid, IWP, Diamond, Neovius) are characterized by dimensions of 8×8×8 mm^3^, subjected to quasi-static uniaxial compression. The top face is displaced, the bottom face is fixed, and the lateral faces are free. Volume fractions vary between 10% and 55%. Meshes are uniform tetrahedral, generated using FlattPack, HyperMesh, and Gmsh, with a discretization parameter μ=35 to 65, resulting in approximately 300,000 elements. The study [37] concludes that incorporating fillet-shape-inspired TPMSs into metal cubic lattice structures significantly enhances their mechanical strength-to-weight ratio. Using AA6082 aluminum alloy and lost PLA casting, the researchers fabricated three lattice variants with varying densities and fillet geometries. Static and dynamic compression tests, both flat and wedge, revealed that TPMS fillets induce beneficial triaxial stress states, outperforming traditional fillet-free designs.

These collective findings underscore the mechanical superiority of TPMS-based architectures over conventional structural designs.

#### 2.4.2. Mass Transfer

**Figure 5:**
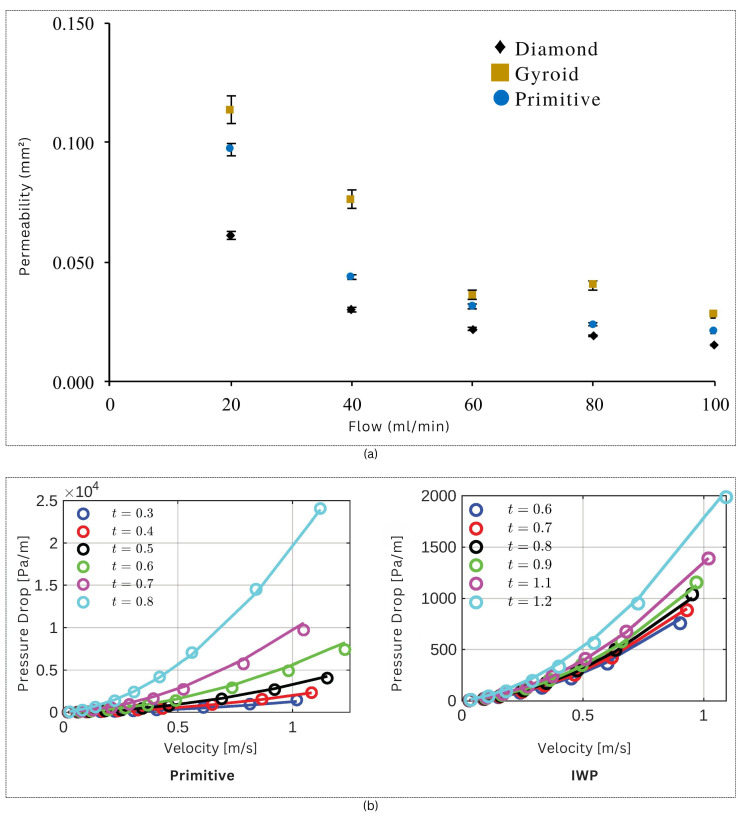
(**a**) Permeability of TPMS scaffolds with respect to flow rate. Reproduced from [41], licensed under CC BY 4.0. (**b**) Comparison between computed and CFD simulations of pressure drop as a function of velocity between Primtive and IWP structures. Reproduced from [42], licensed under CC BY 4.0.

TPMS lattices exhibit superior mass transfer properties because of their their high surface area-to-volume ratio, interconnected pore networks, and smooth, curvilinear pathways that reduce flow resistance and pressure drop. Ref. [40] compared TPMS structures with conventional lattice-based scaffolds, and showed that I-WP and Gyroid surfaces have higher permeability characteristics than other lattices. While most studies have focused on unidirectional flow permeability, applications like tissue engineering require consideration of longitudinal permeability due to cellular growth within porous scaffolds. Experimental results demonstrate that TPMS structures, especially I-WP architectures, exhibit significantly higher longitudinal permeability compared to conventional lattice designs, with radial permeability values measuring roughly 50% of longitudinal values in cylindrical scaffold applications. The correlation between porosity and permeability follows established power law and Kozeny–Carman models. The influence of TPMS scaffold geometry on permeability was experimentally validated using MultiJet printed samples with 70% porosity. As shown in Figure 5a, Gyroid structures exhibited the highest permeability across all flow rates, while Diamond consistently showed the lowest, despite identical porosity. These results underscore the critical role of unit cell design in fluid transport behavior, independent of porosity [41]. Ref. [42] further compared Primitive and IWP structures and showed the accuracy of Darcy–Forchheimer models in predicting the pressure drop across TPMS structures with varying porosity and flow velocity. The comparison between Computational Fluid Dynamics (CFD) simulations and Darcy–Forchheimer model predictions shows strong agreement for both Primitive and IWP geometries, with mean errors of approximately 11% and 5%, respectively. The results as shown in Figure 5b show that IWP structure exhibits lower pressure drop than the Primitive design across all flow velocities, indicating superior permeability.

Further investigation by [43] examined fluid permeability in graded TPMS structures, providing additional insights into their transport properties. These collective findings highlight the enhanced permeability performance of TPMS architectures compared to traditional lattice designs across multiple flow conditions.

#### 2.4.3. Thermal Conductivity

**Figure 6:**
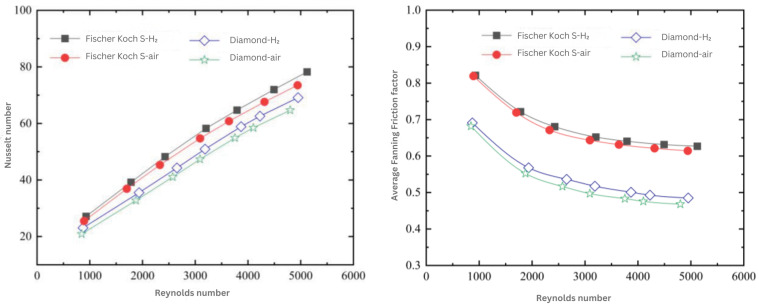
Nusselt number and Average Fanning Friction factor plotted against Reynolds number for Fischer–Koch S and Diamond. Reproduced from [45], licensed under CC BY 4.0.

TPMS-based structures are finding application in heat transfer applications such as heat sinks. Ref. [44] discusses the thermal conductivity of three important types of TPMS lattices, namely Gyroid, Diamond, and Schwarz primitive. This paper explores how thermal conductivity is influenced by the unit cell size and volume fraction. The main findings of the experiment show that the thermal conductivity of TPMS lattices is primarily influenced by the material properties and volume fraction of the specimens examined. However, geometric parameters such as the surface area to volume ratio have a strong influence on conductivity. The study by [45] provides a comprehensive numerical analysis of TPMS-based heat exchangers for aerospace precooling applications. Figure 6 presents the variation of the Nusselt number and Average Fanning Friction factor with Reynolds number for Fischer–Koch S and Diamond structures, revealing that Fischer–Koch S exhibits 13.2–17.6% higher heat transfer performance due to stronger fluid perturbation. These figures collectively underscore the trade-off between thermal enhancement and pressure drop in TPMS geometries.

When examining the impact of surface roughness on thermal performance. The material with a large surface area-to-volume ratio exhibits lower conductivity due to the presence of partially melted material on the surface, which hinders the transfer of heat. Additionally, lattice structures can now be designed to have specific thermal properties by adjusting their parameters, such as the geometry of the cells and volume fraction. The effect of air within the pores on thermal conductivity was studied by [46]. He showed that Schwarz P structures with base conductivity greater than 115 W/mK show air influence within 2%, while Neovius and I-WP lattices with conductivity above 13 W/mK exhibit less than 2% air impact.

## 3. Design and Performance Optimization of TPMS

Although TPMS structures offer many mechanical, thermal, and mass transfer advantages over solid and other lattices, there is still a need to optimize their design to meet the requirements of specific applications. To tailor TPMS structures for specific functional requirements, various advanced design approaches have been developed. These include the Level-Set Method for precise boundary control, relative density grading to adjust material distribution, unit cell size grading for spatial variation in mechanical properties, and multi-morphology strategies that combine different TPMS geometries within a single structure. Together, these methods enable the customization of TPMS architectures for optimized performance in diverse engineering applications. The minimal surfaces can be defined in a number of ways, but implicit function representation, also known as level-set approximation, is the most used. The general form of a level-set function for a TPMS is

(5) ϕ(x,y,z)=f(x,y,z)−c

where ϕ is the level-set approximation, *f* is the mathematical form of TPMSs, and *c* is the threshold value which defines the iso-surface.

The surface’s position, thickness, and material distribution is controlled by the constant *c*. When c=0, the surface divides space into two equal domains. Increasing or decreasing *c* expands or contracts the surface, influencing lattice structure. Solid lattices form by retaining material on one side (ϕ>c) or (ϕ<c), while sheet-based lattices emerge by thickening the surface (−c≤ϕ≤c), as shown in Figure 3. Adjusting *c* allows precise control over density, mechanical properties, and functionality.

### 3.1. Functional Grading

We cannot fully utilize the potential of TPMS structures when they are uniform because certain applications may require more concentrated regions where the stress is high and low concentrated regions with lower stress values, so a gradual change to surface area, material’s volume fraction, or pore size is needed. This concept is commonly known as functional grading. Functional grading can be achieved by spatially varying the level-set constant, like volume fraction, pore size, or surface area, within the Cartesian space, allowing for tailored material properties. Such gradual change offers one of the key advantages of minimizing the stress concentration.

#### 3.1.1. Relative Density Grading

**Figure 7:**
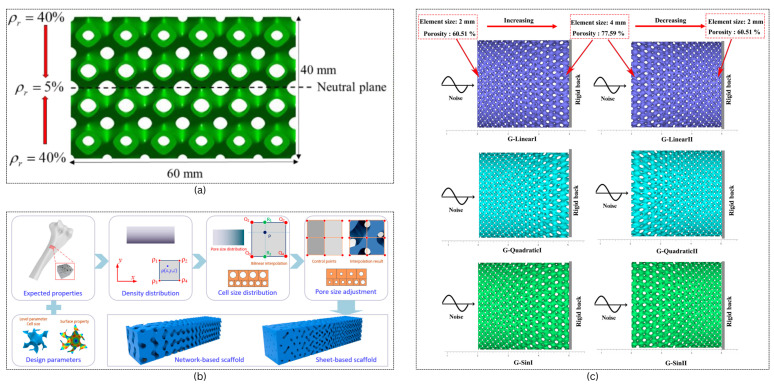
Examples of functionally graded TPMS structures. (**a**) Bilinear relative density grading of a TPMS lattice structure. Reproduced from [48], licensed under CC BY 4.0. (**b**) Method of the generation of a functionally graded structure with programmable pore size. Reproduced from [50], licensed under CC BY 4.0. (**c**) Designed model of the graded gyroid structures sound absorption performance. Reproduced from [51], licensed under CC BY 4.0.

Lattices are recently being used where weight reduction is the priority. The mechanical properties of such structures depend upon the relative density and their topology [47]. The simplest approach to create a functionally graded lattice is by altering the constant c, which directly affects the thickness of the sheets. Ref. [48] applied a bilinear gradation scheme through the thickness of a TPMS-based beam as shown in Figure 7a. The design embeds lower-density cells near the neutral axis and higher-density cells toward the top and bottom faces, where tensile and compressive stresses peak.

#### 3.1.2. Unit Cell Size Grading

Unit cell size grading in TPMS structures is a powerful design strategy used to spatially vary the geometry of the lattice while maintaining consistent relative density. This approach enables tailored mechanical, thermal, and fluidic performance across a structure.The second way of grading is by changing the unit cell size and keeping the relative density constant. This can be achieved by changing *L* in the level-set equations. The unit cell shape and size of a TPMS expression is controlled by α, β, and γ. These parameters control the unit cell size and shape. To obtain a cell size gradient, the α, β, and γ should be defined as functions in all directions.

(6) $$\left\{\begin{array}{c} u_{\left(x\right)}=\alpha_{\left(x,y,z\right)}\cdot x\\ v_{\left(y\right)}=\beta_{\left(x,y,z\right)}\cdot y\\ w_{\left(z\right)}=\gamma_{\left(x,y,z\right)}\cdot z \end{array}\right.$$

To avoid distortion in structure the following relation needs to be satisfied [49]

(7) $${\dot{u}}_{x}={\dot{v}}_{y}={\dot{w}}_{z}$$

Ref. [50] introduce a parametric design method for TPMS-based scaffolds that enables simultaneous control over relative density and pore size distribution. Figure 7b presents visual comparisons of solid and sheet-based gyroid scaffolds with programmable pore sizes, highlighting how spatial variation in unit cell size and level-set parameters can produce smooth gradients in porosity while maintaining consistent pore volumes.

Ref. [51] developed a design methodology for graded TPMS structures tailored for broadband sound absorption and mechanical strength. Figure 7c presents the CAD models of gyroid structures with three types of graded element size distributions—linear, quadratic, and sine—along two porosity orientations (Type-I and Type-II).

### 3.2. Hybridization

We can create a lattice structure with two or more types of unit cells to meet our specific structural or other requirements. The transition between the unit cells can be defined mathematically. For a smooth transition, a new phase between two adjacent minimal surfaces needs to be employed. Ref. [52] proposes two novel approaches of hybridization: sigmoid function and Gaussian radial basis function.

#### 3.2.1. Sigmoid Function

One is the sigmoid function method, which is applied to simple transition boundary cases, given by

(8) $$\gamma \left(x,y,z\right)=\frac{1}{1+e^{kG(x,y,z)}}$$

where G(x,y,z) represents the spatial coordinate function that defines the shape of the transition between different regions, and *k* controls the width of the transition.

#### 3.2.2. Radial Basis Function

**Figure 8:**
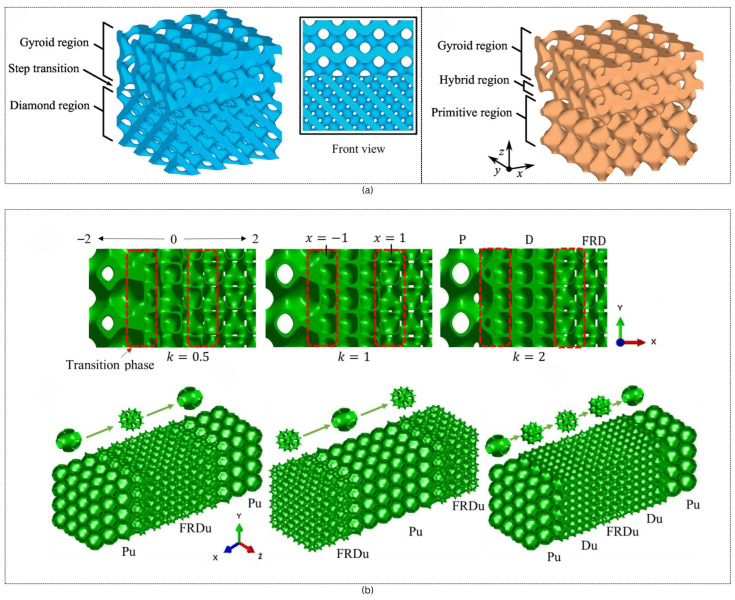
Demonstration of hybridization of TPMSs. (**a**) Hybrid TPMS lattices using sigmoid transitions: sharp Diamond–Gyroid interface and smooth Primitive–Gyroid blend along the build direction. Reproduced from [53], licensed under CC BY 4.0. (**b**) Illustration of hybrid lattice structures comprising Primitive, Diamond, and F-Rhombic Dodecahedron for different values of the topology transition parameter *k* at 20% relative density. Reproduced from [48], licensed under CC BY 4.0.

The second function is the Gaussian radial basis function (GRBF), which can be applied to more generic cases. The sigmoid function method is a practical and effective approach to merging two cellular structures, but it may face challenges when dealing with intricate and compound transition boundaries. The GRBF method has a higher capacity for transitioning between different morphological forms with compound boundaries. Figure 8 provides the visual of the use of sigmoid transition and various hybrid structure geometry.

### 3.3. Computational Methods

Computational methods are being developed to optimize structure in a variety of engineering and industrial applications. The most-used tool in these techniques is TOD. This optimization technique is conducted to determine the most effective structural layout. It is a branch of optimization that is different from size and shape optimization. Like other optimization methods, it involves constant parameters such as applied loads and material properties, as well as an objective function and constraints that vary for each problem. The very first research on topology optimization was conducted by [54].

Topology optimization has proven to be an effective tool for identifying optimal design concepts in the early stages of the design process. It is extensively used for lightweight structural design in the automotive and aerospace sectors and also finds applications in civil engineering, materials science, and biomechanics.

A general topology optimization problem is to find a material distribution that minimizes an objective function *f* with several constraints. The general form is formulated as follows:

minf(u(χ(x)),χ(x))

where u(χ(x)) is the displacement field that depends on the material distribution χ(x), and χ(x) is the design variable at each point *x*, representing whether material is present (χ(x)=1) or absent (χ(x)=0) at point *x*.

This is subject to

$$\begin{array}{cc} g(u(\chi (x)),\chi (x))\; & \leq 0\\ K_{\omega }\left(\chi \left(x\right)\right)u_{\omega }\left(\chi \left(x\right)\right)\; & =P(x) \end{array}$$

where $K_{\omega }\left(x\right)$ is the stiffness matrix of the material in region ω(x), u(x) is the displacement field at point *x*, and P(x) is the external load at point *x*.

Ref. [55] introduced the formulation of topology optimization (TO)-driven functional grading of TPMS lattices in a compliance problem. The paper also implemented sigmoid filtering to enforce volume fractions equal to zero only when lower than the bound.

$$\left\{\begin{array}{c} \min_{\rho ,\;d}\;F^{T}d\\ s.t.\;\left\{\begin{array}{c} K(\rho )\;d=F,\\ V\left(\rho \right)\leq \hat{V},\\ \epsilon \;1^{T}\leq \rho \leq u_{b}\;1^{T} \end{array}\right. \end{array}\right.$$

where F contains the external forces, d is the displacement vector, ϵ is a small number, and $1^{T}$ is a vector of ones.

The homogeneous stiffness matrix for each finite element e is defined and used in aforementioned compliance problems for optimal grading, where $f_{ij}\left(\rho \right)$ become weights which modulate the Voigt form of an elastic matrix, whose base entries are base material constants $c_{\alpha }$, scaled by trigonometric level-set fields $f_{i}$.

$$C_{e}=C_{e}\left(\rho_{e}\right)=\left[\begin{array}{cccccc} f_{11}c_{11} & f_{21}c_{21} & f_{31}c_{31} & 0 & 0 & 0\\ f_{21}c_{21} & f_{22}c_{22} & f_{32}c_{31} & 0 & 0 & 0\\ f_{31}c_{31} & f_{32}c_{31} & f_{33}c_{33} & 0 & 0 & 0\\ 0 & 0 & 0 & f_{44}c_{44} & 0 & 0\\ 0 & 0 & 0 & 0 & f_{55}c_{44} & 0\\ 0 & 0 & 0 & 0 & 0 & f_{66}c_{66} \end{array}\right],$$

(9) $$\begin{array}{c} f_{1}=\sin(\gamma x)\cos(\gamma y)+\sin(\gamma y)\cos(\gamma z)+\sin(\gamma z)\cos(\gamma x)\pm \kappa , \end{array}$$

(10) $$\begin{array}{cc} f_{2}=\; & \left[\sin(2\gamma x)\sin(\gamma z)\cos(\gamma y)+\sin(2\gamma y)\sin(\gamma x)\cos(\gamma z)+\sin(2\gamma z)\sin(\gamma y)\cos(\gamma x)\right]\\ & +0.2\left[\cos(2\gamma x)\cos(2\gamma y)+\cos(2\gamma y)\cos(2\gamma z)+\cos(2\gamma z)\cos(2\gamma x)\right]\pm \kappa , \end{array}$$

(11) $$\begin{array}{cc} f_{3}=\; & \sin(\gamma x)\sin(\gamma y)\sin(\gamma z)+\sin(\gamma x)\cos(\gamma y)\cos(\gamma z)\\ & +\cos(\gamma x)\sin(\gamma y)\cos(\gamma z)+\cos(\gamma x)\cos(\gamma y)\sin(\gamma z)\pm \kappa . \end{array}$$

where γ represents the size of the period of the lattice (unit cell size) and κ shows the thickness of the lattice; Equations (9)–(11) are higher-order generalizations of surfaces defined in Equations (1)–(4), which are more classical TPMS equations.

#### 3.3.1. Level-Set Methods

Level-set methods implicitly define geometry using a level-set function, representing it as the zero level-set of a higher-dimensional function. This approach allows for smooth shape modifications, including the merging and splitting of material regions, without the need for explicit boundary tracking. A highly efficient implementation is the level-set method introduced by [56], which represents interfaces as level-sets of a higher-dimensional function. The key advantage of this implicit representation is its natural ability to accommodate topological changes.

#### 3.3.2. Density-Based Methods

The density-based methods take a characteristic function or an indicator function to impose solid material/void material in the structure. This approach connects material distribution to element density variables using interpolation models, such as the Solid Isotropic Material with Penalization (SIMP) model [57], and the Rational Approximation of Material Properties (RAMP) model [58]. O Sigmund wrote 99-line code in MATLAB to implement this SIMP model [59]. The code aims to minimize compliance (maximize stiffness) while maintaining a specified volume fraction. It employs the FEM to analyze structural behavior under applied loads and iteratively adjusts the material distribution using the optimality criteria (OC) method.

#### 3.3.3. Geometric Projection-Based Methods

Recent methods in structural optimization aim to achieve designs with specific high-level geometric features without requiring re-meshing. These approaches facilitate the embedding of primitive-shaped components, the design of stocks of materials, the control of structural dimensions, and the guarantee of CAD compatibility. They often use fewer variables, which benefits gradient-free optimization. These methods often use implicit functions in their formulation, but their focus is on their ability to control high-level geometric features, a capability not inherent to level-set methods, in general. Ref. [4] proposed the name feature mapping for these methods.

#### 3.3.4. Genetic Algorithms

Density-based methods use continuous design variables, thus converting the problem into a continuous optimization problem. The resulting elements can be of intermediate density, so post-processing steps like binarization are required, making it hard to guarantee that the final structure is truly optimal. In contrast, a relatively different technique called genetic algorithms treats the discrete problem directly and explores the design space by simulating biological processes like mutation, crossover, and selection [32,60,61]. Their main advantage is that they reduce computational effort by avoiding gradient calculations. Instead, they search for the optimal topology by evaluating only the objective function and the constraints.

### 3.4. Advancement in Topology Optimization Methods

Numerous studies have explored data-driven approaches to tackle the problem of topology optimization, notably [62,63], who address challenges such as inaccurate or sub-optimal predictions and propose novel solutions. Ref. [64] further presents a critical instant analysis method to handle force location uncertainty in lightweight structure design. On the computational side, works by [65,66] demonstrate the use of GPU-based parallel processing to accelerate large-scale topology optimization while achieving high resolution and accuracy.

Recently, machine learning has emerged as a promising tool for reducing the computational cost associated with topology optimization. Ref. [67] applies generative models like VAEs and GANs, using loading and displacement boundary conditions as inputs to predict optimized structures. Similarly, Ref. [63] employs PCA and a fully connected neural network to map loading configurations to optimal topologies. Recently, numerous studies have explored the use of neural networks as surrogates for finite element analysis (FEA), employing physics-informed neural networks (PINNs) [68], or for predicting the performance of the objective function [69].

## 4. Topology Optimization of TPMS Structures

Lattice structures like TPMSs require multi-scale optimization, which consists of optimizing the microstructure and macrostructure for the required objective function. This kind of TO is called Multiscale Topology Optimization (MTSO) [70]. Currently there is a lack of simple and efficient techniques that can optimize both the structure at both macro and micro levels. One approach is two-scale optimization [71,72,73,74] which concurrently optimizes the structure at the micro and macro level. Ref. [75] proposes MulaTOVA, a multi-lattice topology optimization framework integrating variational autoencoders and neural networks to concurrently optimize macro- and mesoscale structures.

Recently ref. [76] proposed topology optimization of TPMS-based structures based on the density method. He introduced a new approach to design functionally graded cellular structures using variable-density gyroid geometries. The approach ensures continuity and connectivity of gyroid structures, determines mechanical properties through a density-based scaling law, and integrates this into the optimization algorithm for optimal density distribution.

**Figure 9:**
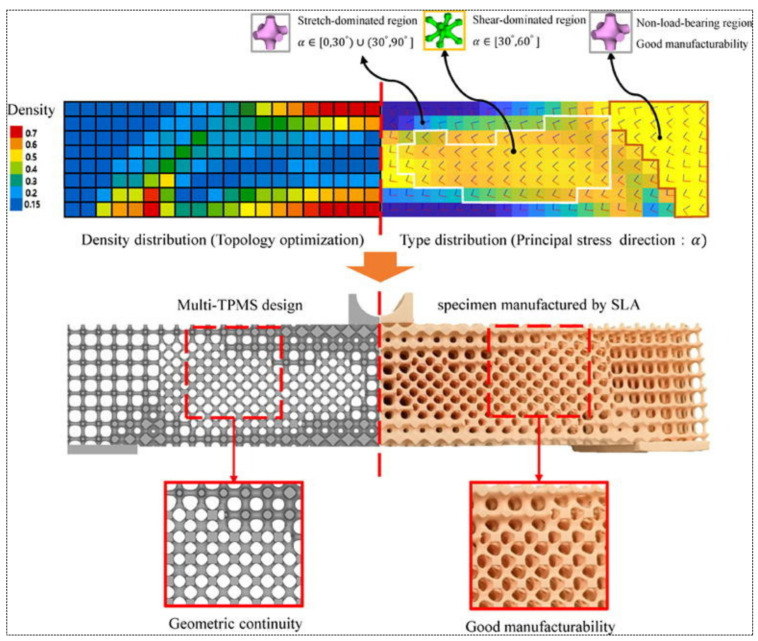
Multi-TPMS topology optimization. Reproduced from [77], licensed under CC BY 4.0.

To design a Multi-TPMS Structure using topology optimization, [77] proposed a method. By assigning specific lattice cells and their corresponding densities to different stress regions, the distinct characteristics of each cell type are effectively leveraged to enhance the overall mechanical performance. To smoothen the transition between different lattices, an improved Sigmoid function was used as shown in Figure 9.

## 5. Manufacturing Methods for TPMS-Based Structures

The design and fabrication of porous structures, especially TPMS structures, has attracted great interest because of their remarkable mechanical, thermal, and biological performance. Nevertheless, the conventional manufacturing techniques have become major hindrances in achieving these complicated structures. Common manufacturing processes in the form of machining, casting, and plastic deformation are inherently limited by the need for the accessibility of tools and the removal of molds. Fabrication of TPMSs with these techniques is made nearly impossible due to the continuous internal pores, curved interiors, and connectivity typical of TPMS structures. Despite lost-wax casting-like techniques in which sacrificial dies are employed, the procedure itself is rendered laborious and unable to produce the geometric accuracy or scalability needed for contemporary applications. A number of unconventional methods such as salt leaching, gas foaming, freeze-drying, and electro spinning have been utilized for production of porous structures. Although these methods are able to create random porosity, they have no control over key structural parameters like pore size, shape, and inter-connectivity. As a result, the final structures are stochastic in nature, which delimits their consistency in performance and their design adaptability. The emergence of AM has transformed the way of manufacturing intricate porous structures. AM technologies have successfully eliminated the limitation of the geometric complexity that constrained traditional methods by building the material layer by layer from digital models. This new advance has made it possible to accurately create periodic porous structures like TPMS structures. With additive manufacturing, it is possible to create structures by designing controllable pore geometry and high reproducibility, achieving a closer connection between computational design and physical implementation. There are a number of additive manufacturing methods that exist for the fabrication of TPMS structures; hence, in this section, each of these methods will be discussed in detail.

### 5.1. Stereolithography (SLA)

SLA, one of the earliest AM technique, creates 3D parts by polymerizing photosensitive liquid resin in a photopolymerization reaction by ultraviolet (UV) light or alternative sources. The technique uses a laser or a projector to polymerize each new layer and produce printing precision and a smooth surface finish [14]. This technique, by the utilization of UV light and photopolymer sensitive materials, provides the highest resolution of all the AM methods. Because of this property, SLA has become increasingly popular for the construction of TPMS-based components because of their very high precision and control [78].

The development of biodegradable structures using SLA, first reported by [79], marked the beginning of its biomedical applications. Since then, SLA has proved strong potential in fabricating TPMS-based bioceramic scaffolds with precise geometrical control. For example, using tricalcium phosphate, SLA-enabled gyroid structures achieved tunable porosity, pore size, and mechanical strength closely resembling cancellous bone. This high-precision fabrication not only increased structural fidelity but also promoted superior cell proliferation in contrast to conventional grid-like scaffolds [80]. A key practical consideration for such ceramic-based SLA is the significant ’sintering shrinkage’ that occurs during post-processing. To ensure final dimensional accuracy, the original digital models were scaled up by a factor of 1.2 to compensate for this effect.

For the biomedical area, micro-stereolithography (MSTL) has been employed to create precisely biodegradable polymer scaffolds. For example, MSTL has successfully imprinted 65% porous scaffolds of polypropylene fumarate (PPF) that have undergone surface modification by biomimetic apatite and RGD peptide coatings to improve osteoblast adhesion and growth for exhibiting significant potential towards bone tissue engineering [81]. Following these results, later work again confirmed that TPMS gyroid lattices fabricated by SLA showed higher mechanical performance than the stochastic structures and approached the elastic moduli and yield strengths for natural cancellous bone, thus confirming the potential of the SLA for the fabrication of bone-mimicking porous structures having controllable mechanical performances [82]. Aside from that, the TPMS geometries such as Gyroid, Diamond, Primitive, Neovius, Fischer–Koch S, and Split-P have also been successfully fabricated by the SLA. Comparative investigations by testing the structures under compressive loading revealed that the highest strength corresponded to the Gyroid structure, followed closely by Neovius, despite the thinner walls of the latter [83]. Subsequently, research extended this insight further by preparing Schwarz Primitive and Gyroid TPMS scaffolds by the SLA technique using dental resin and underscoring the fact that wall thickness significantly impacts the elastic modulus and compressive strength. The outcome also established a power-law dependence between relative density and elastic modulus for both structures while validating the accuracy and malleability of the SLA technique for the preparation of functional TPMS scaffolds [84].

### 5.2. Digital Light Processing (DLP)

DLP is an additive manufacturing technology that is similar to SLA because both technologies involve photopolymerization of light-sensitive resin layer by layer using light. The primary distinction lies in the fact that where a laser is employed by SLA to cure individual layers progressively by beam scanning, DLP cures the entire layer instantaneously using a digital projector. The result is DLP being comparatively quicker than SLA but maintaining high detail surface finish and accuracy. The DLP technology has become increasingly employed for the construction of TPMS-based structures due to its capabilities for achieving intricate geometries of superior resolution and structural homogeneity. With this technique, the successful construction of Yttria-stabilized tetragonal zirconia polycrystal (3Y-TZP) ceramic structures of Diamond-type TPMS architectures has been realized, achieving a high relative density of 98.6% and flexural strength of 937 MPa while retaining superior thermal insulation capabilities [85]. Likewise, hydroxyapatite scaffolds reinforced by bredigite (BR) have been synthesized using desktop-level DLP printing to show enhanced bioactivity and higher degradation rates favoring bone regeneration [86]. DLP has also been employed for the fabrication of visible-light-cured hydrogel scaffolds of Primitive-type TPMS morphologies for achieving sustained release of oxygen for long-duration cell viability and improved bone repair in vivo [16]. For applications outside the biomedicine field, DLP-printed gyroid TPMS structures have been employed for studying permeability behavior that manifests a strong dependence of radial flow and fluid transport qualities on porosity and viscosity [87]. Moreover, DLP-created Mg-doped wollastonite scaffolds for s-Gyroid, s-Diamond, and IWP morphologies have shown an enhanced four-fold increase in their compressive strength and elevated release of Mg ions while retaining higher Diamond and Gyroid structures for excelling in osteogenic differentiation and bone ingrowth. DLP-based TPMS-manufactured ceramics also encounter the problem of volumetric shrinkage in post-process sintering, which seriously affects dimensional accuracy. This shrinkage is often anisotropic. It was recorded that the final porosity of the sintered scaffolds was 3–7% lower compared to the designed CAD value due to greater macro-pore compaction [88].

### 5.3. Selective Laser Melting (SLM)

SLM employs a high-power laser to fully melt metallic powders, enabling the production of dense and complex TPMS lattices. It offers excellent precision and mechanical strength, making it effective for both biomedical and structural components. The research has also revealed that CP–Ti gyroid scaffolds with 68–73% porosity showed elastic moduli (1465–2676 MPa) and yield strengths (44.9–56.5 MPa) close to trabecular bone while maintaining ductility up to 50% strain ideal for implants [17]. Further research shows early-stage osteogenesis is promoted by bulk porosity, whereas surface roughness supports long-term remodeling [89]. Beyond biomedicine, laser powder bed-fused gradient TPMS lattices achieved a 19.5% rise in energy absorption and a 28.5% reduction in peak stress, highlighting their potential in impact protection and lightweight applications [90]. SLM-fabricated TPMS structures face the critical issue of thermal shrinkage and residual stresses. Mitigating the resulting warping and maintaining dimensional accuracy typically requires the use of heated build plates and optimized laser scan strategies.

### 5.4. Selective Laser Sintering (SLS)

SLS fuses polymer powders layer by layer with a laser beam, enabling support-free fabrication and complex geometries, though with slightly rougher surfaces and lower precision than SLM. It is well-suited for TPMS fabrication due to its accuracy and ability to produce intricate porous structures. Studies show sheet-type TPMS lattices deform by stretching and exhibit superior stiffness and strength, while ligament-based designs are bending-dominated and ideal for energy absorption [91]. Testing across 55 polymer lattices confirmed that sheet-type TPMSs deliver the best mechanical performance, even at low densities [19]. SLS has also produced gyroid-based thermal insulation materials with low thermal conductivity (0.033 W/m·K) and high resistance (0.606 m^2^·K/W), suitable for lightweight insulation [92]. SLS is also subject to thermal shrinkage, which occurs when the powder that is partially melted cools down and gets denser after sintering. Pre-heating control of the powder bed is necessary to achieve accurate part density, and to mitigate dimensional errors.

### 5.5. Electron Beam Melting (EBM)

EBM is a type of additive manufacturing that applies a high-energy electron beam to melt metal powders sequentially inside a vacuum chamber. The level of temperatures possible by the electron gun exceeds that of laser-based equipment so that EBM is suited for the processing of strong and highly reactive metal alloys such as Ti-6Al-4V but applies only to conductive metals. Recent studies confirm the success of EBM for biomedical applications. For instance, gyroid-shaped TPMS structures fabricated by EBM in both Wafer and Melt themes exhibited comparable mechanics, reaching human cortical bone-like elastic modulus for strong bone implant potential [18]. EBM was also utilized by another research work to create scaffolds of Ti-6Al-4V where it showed that the optimum unit size minimized the number of crack initiation sites for enhanced fatigue life of greater than $10^{6}$ cycles without comptomising porosity. EBM benefits from the use of a high preheating temperature, which is a key advantage for mitigating thermal shrinkage and avoiding thermal cracks [93].

### 5.6. Fused Deposition Modeling (FDM)

FDM is a cost-effective additive manufacturing technique that builds parts by depositing thermoplastic filament layer by layer. It offers material flexibility and operational ease but produces lower surface finish and weaker interlayer bonding than resin-based methods. Its ability to form lightweight, porous geometries makes it suitable for TPMS fabrication, where mechanical behavior can be tuned via material type, infill, and print orientation. TPMS structures such as Diamond, Schwarz, and Gyroid printed in medical-grade PLA showed cancellous bone-like properties, good biocompatibility, and enhanced cell growth [20]. Comparisons of Schwarz-P grids made from PLA and ABS revealed 30% higher peak stress and lower compressibility in PLA, confirming FDM’s reliability for polymer TPMS fabrication [94]. Under hydrothermal aging, Gyroid and Diamond forms retained over 90% strength, while Primitive and IWP degraded faster due to weak interlayer bonding [95]. FDM requires precise control of the thermal environment through heated plates and chambers to prevent thermal shrinkage from causing geometric defects such as warping and interlayer delamination. Overall, FDM remains versatile and affordable but requires surface and bonding improvements for advanced applications.

### 5.7. Other Special Fabrication Methods

The construction of TPMS structures requires very accurate manufacturing methods because of their highly detailed geometries and thin lattice structures. A number of sophisticated AM methods have been designed for this purpose and offer distinct advantages in terms of resolution, usable material base, and structural soundness.

### 5.8. Projection Micro-Stereolithography (PµSL)

PµSL is a UV-light lithography technology that creates high-precision polymer micro-lattices by writing UV light through a variable mask to polymerize photosensitive resin in a layer-by-layer manner. This allows for the realization of intricate TPMS geometries like the Gyroid or shell-like structures in superb surface quality. But defects that arise from the process such as non-uniform wall thickness or small geometric variations will impact the mechanical performance. Research indicates that such defects notably impact the compressive strength and deformation modes while noting the importance of accurate calibration and process tailoring [96].

#### 5.8.1. Two-Photon Polymerization (2PP)/Direct Laser Writing (DLW)

2PP is a very precise 3D micro-fabrication technique that has the capability of fabricating features of less than 100 nm. It causes polymerization only at the location of the femtosecond laser focus such that true 3D shaping occurs without the requirement for masks. TPMS structures such as Schwarz P, Gyroid, and Neovius have successfully been fabricated by the 2PP procedure so that highly precise micro-scaffolds with smooth surfaces have been completed. The structures have been found to have excellent mechanical performance—with high modulus and energy absorption—and may also be further strengthened by thin covers of ceramic for further greater strength [97]. For tissue engineering purposes, the 2PP procedure produces biocompatible scaffolds that are customizable and have controllable porosity, stiffness, and permeability such that natural extracellular matrices are accurately replicated. The procedure also has co-cultivation design and permeability regulation by scanner speed and exposure such that the procedure is a very promising route for the future generation-level biomedical TPMS scaffolds [98]. 2PP requires fine control over power and dosage in order to mitigate shrinkage due to photopolymerization, which is the major factor limiting nanoscale dimensionality and imposing residual stresses on printed micro-features.

#### 5.8.2. Robocasting

Robocasting or Direct Ink Writing (DIW) is a type of extrusion-based AM that accumulates viscous ceramic or polymer pastes in a sequential building process to generate accurate 3D structures. It has very good control over porosity and shape and is very suitable for building TPMS-based bone scaffolds. Recent studies successfully fabricated hydroxyapatite Gyroid and Fischer–Koch S (FKS) TPMS scaffolds by robocasting with photopolymerization for high dimensional accuracy and bioactivity [21]. The FKS scaffolds were found to have 32% greater strength and 49% higher energy absorption than the Gyroid structures and are promising for dense bone regeneration [99]. Robocasting structures undergo significant sintering shrinkage, up to approximately 21%, which should be accounted for during initial design efforts to ensure target dimensions are met. This is usually anisotropic, with the Z-axis showing a measurably greater reduction due to layer-based variability and extrusion pressure effects. Different techniques are carefully summarized in Table 1 for TPMS.

## 6. Applications of TPMS Lattice Structures

TPMS structures are used in many engineering and medical applications because of their special mechanical properties, high surface area-to-volume ratio, and efficient thermal dissipation. TPMS structures are widely employed wherever high-strength and lightweight materials are required for optimal thermal or biological performance. Their interconnected and uniform pores make them particularly suitable for applications involving control of fluid flow, thermal management, and high-strength structure. The following sections will elaborate on these applications in detail, explaining how TPMS structures enhance functionality in various ways.

### 6.1. Heat Sink

The rapid evolution of computing technology, which is powerful, cheaper, and faster, has transformed technology, leading to immense innovations in society [100]. However, this progress has introduced substantial thermal management challenges, necessitating innovative cooling solutions to ensure reliable device performance [101].

#### 6.1.1. Conventional Heat Sinks

Heat sinks (HSs) have been widely adopted in electronics due to their simplicity, reliability, and cost-effectiveness. Traditional HS designs primarily feature flat-plate or pin-fin structures, but recent studies have demonstrated that porous micro-channel heat sinks (MCHSs) with configurations like trapezoidal or sandwich designs exhibit superior thermal and hydraulic performance, particularly at higher Reynolds numbers [10,102].

#### 6.1.2. Emergence of TPMS-Based Heat Sinks

TPMS-based cellular structures are a promising method for the optimization of heat sinks. Because of their inherently high surface-to-volume ratio, smooth connectivity, and ability to tailor thermal conductivity, TPMS architectures can improve the efficiency in dissipating heat. Computational studies have demonstrated that mapping of porous TPMS structures such as gyroid sheets is able to achieve higher thermal conductivity and better temperature control by topology optimization [7,9].

**Figure 10:**
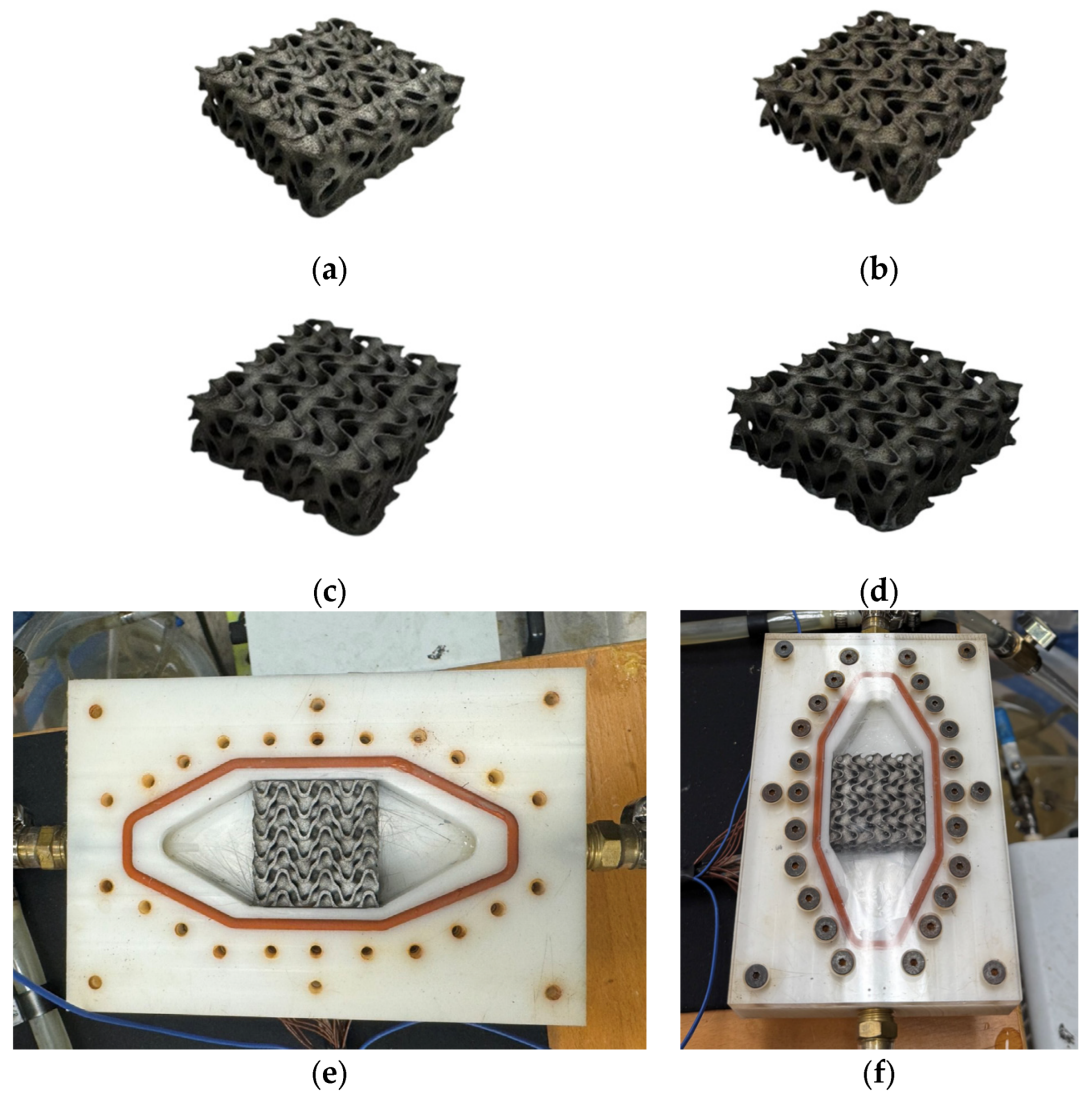
Experimental FKS-TPMS heat sink samples with varying porosities: (**a**) porosity 0.6, (**b**) porosity 0.7, (**c**) porosity 0.8, (**d**) gradient porosity (0.6–0.8), and (**e**,**f**) experimental test section. Reproduced from [103], licensed under CC BY 4.0.

Recently, a new experimental study has been conducted on the Fischer–Koch S (FKS) TPMS heat sink for different porosity arrangements, such as 0.6, 0.7, 0.8, and gradient porosity (0.6–0.8) (Figure 10). It was observed that low-porosity and gradient designs caused a significant enhancement of the convective heat transfer mechanism, which resulted in an improvement of up to 54.46% in the heat transfer coefficient and a reduction in thermal resistance of 31.8% compared with high-porosity geometries. The FKS geometries also demonstrated lower surface temperatures (up to 19.5%) and better temperature uniformity at various mass flow rates compared to conventional straight-channel heat sinks (SCHSs). These observations lead to the conclusion that optimized geometries of TPMS will not only increase surface interaction but will continue cooling efficiently under dynamic flow conditions [103].

These findings highlight the potential of TPMS-based heat sinks to outperform traditional solutions, offering enhanced cooling performance for next-generation electronics.

#### 6.1.3. Optimization of TPMS Structures for Thermal Efficiency

A novel methodology in the design and optimization of TPMS-based porous shell structures for enhanced heat dissipation efficiency is presented in [8]. The authors presented the determination of wall thickness and period in such porous materials, avoiding re-meshing. The proposed structures were compared, at identical volume constraints, heat source size, and average wall thickness, to standard heat sink designs, which include plug and V-shaped heat sinks. The optimized TPMS-based structures demonstrate higher heat conduction efficiency by showing lower thermal compliance and reduced maximum temperatures.

This trend is further supported by the experimental validation from the FKS-TPMS study, which indicates that as porosity decreases from 0.8 to 0.6, both the Nusselt number and convective heat transfer coefficient increase considerably. The gradient configuration (P678) achieved a more uniform temperature distribution, emphasizing the influence of topological structure and porosity tuning on optimized thermal and hydraulic performance in TPMS-based systems [103].

This research collectively establishes a framework for topology and geometry optimization in heat dissipation applications. It illustrates how TPMS-based porous structures can perform better than lattice and conventional heat sinks, providing increased computational and thermal efficiency. The proposed techniques have wide-ranging implications for applications in the automotive, aerospace, and electronics sectors, where lightweight construction and effective heat dissipation are essential.

#### 6.1.4. Summary of TPMS-Based Heat Sinks Performance

To consolidate the discussed findings, the following Table 2 presents a comparative summary of different TPMS-based heat sink designs. It highlights their performance in terms of thermal compliance, temperature reduction, and structural efficiency under different design optimizations.

### 6.2. Interpenetrating Phase Composites

Interpenetrating phase composites (IPCs) are multiphase materials in which each phase forms a continuous, interconnected network throughout the microstructure [104]. IPCs differ from traditional composites with a matrix reinforced with fibers, particles, or whiskers in that they have co-continuous phases. Therefore, this often contributes to an overall enhancement in properties [105].

#### 6.2.1. Mechanical Advantages of IPC Structures

Their interpenetration is responsible for the enhanced mechanical properties of IPCs, enabling higher elastic modulus, energy absorption, and compression strength compared to conventional composites. It has been found that the syntactic foams treated with silane, when compared to syntactic foams of the same composition, have 50% more absorption of energy per unit volume, as well as 42% enhancement in plateau stress [106]. Another study also reported that the energy absorption ability of IPCs was up to 720 kJ/kg due to the interphase interactions, primarily governed by shear between the soft and hard phases. Ref. [107] showed that the connected structure of reinforcements affects the elastic and plastic behavior of aluminum composites. Ref. [108] demonstrates that IPCs reinforced with lattice networks exhibit elastic moduli 1–3 times higher than conventional particles or fiber-reinforced composites and achieve near-isotropic behavior through tailored reinforcement geometry.

#### 6.2.2. Role of Additive Manufacturing and TPMS Integration

IPCs have an edge over properties from different kinds of composites, but there is also a lot of research related to further optimizing these IPCs. The emergence of AM has revolutionized the manufacturing of complex structures, including TPMS structures. TPMS materials are used as reinforcement in these structures, and matrix material is the subtraction of the lattice structure from the solid material.

#### 6.2.3. Performance Comparison of TPMS-Based IPCs

The study [11] discusses the design, manufacturing, and properties of TPMS-based aluminum–alumina IPCs. It was found that the ceramic volume fraction, number of unit cells, and the type of TPMS structure greatly affects the compressive strength of ceramic structures. Comparing geometries, Diamond and Gyroid IPCs have, on average, 6.79 ± 3.10% higher energy absorption than Primitive IPCs. An increase in the ceramic volume fraction led to a linear increase in compressive strength. Increasing the number of unit cells from 2 × 2 × 2 to 3 × 3 × 3 results in an 18.65% improvement in compressive strength. Compared to aluminum alloy, the combined structure of aluminum alloy and alumina TPMS has approximately 6% more compressive offset stress.

**Figure 11:**
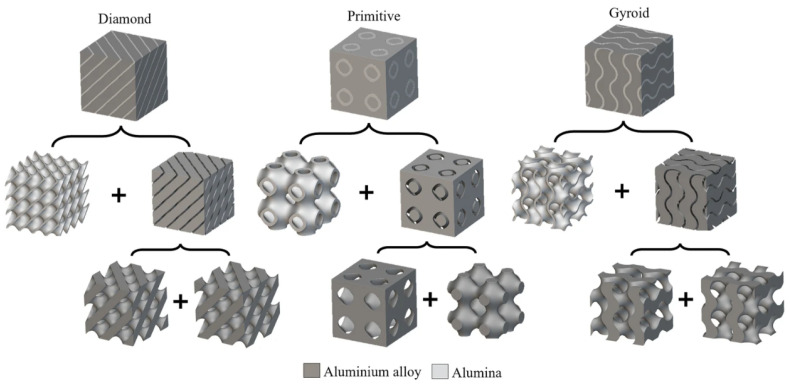
Decomposed IPCs with 20% volume fraction and 2×2×2 unit cells. Reproduced from [11], licensed under CC BY 4.0.

Primitive IPCs possess distinct characteristics relative to Gyroid and Diamond IPCs. Due to the fact that Primitive TPMSs consist of a closed-cell structure, the IPC consists of three spatially independent phases: the aluminum oxide structure, aluminum contained within it, and aluminum enclosing it, as shown in Figure 11. This division means that the two phases of aluminum are unrelated to one another, with neither chemical nor physical bonding. In contrast, although Gyroid and Diamond IPCs consist of three phases that are spatially independent, their mechanical engagement enhances structural performance by raising plateau stress and energy absorption. Table 3 depicts a discreet overview of recent TPMS applications in IPCs.

### 6.3. Biomimetic Scaffolds

TPMS structures are increasingly used in biomimetic scaffolds due to their high surface area, interconnected porosity, and mechanical tunability. These properties make them ideal for orthopedic, dental, and chondral applications, where they can mimic natural bone architecture, support tissue regeneration, and enhance cell attachment and nutrient transport.

#### 6.3.1. Orthopedic Applications

Tissue engineering has made significant progress in developing biomimetic scaffolds, which enable new tissue development by mimicking the structure and functionality of biological tissue. These scaffolds support cell attachment, growth, and healing, making them highly useful in bone and cartilage repair. Among all kinds of scaffolds, TPMS-based scaffolds have attracted much attention due to their tunable mechanical properties, high porosity, and permeability, with advantages for good transport of nutrition and tissue incorporation. Their surface morphology is most similar to that of trabecular bone and enables the cells to grow and develop new tissue more efficiently. Recent advancements in AM have made it possible to fabricate complex structures, thus allowing the production of personalized implants designed for individual patients [13]. Such characteristics make TPMS-based biomimetic scaffolds a suitable candidate for bone repair, orthopaedic implants, and other medical applications.

**Figure 12:**
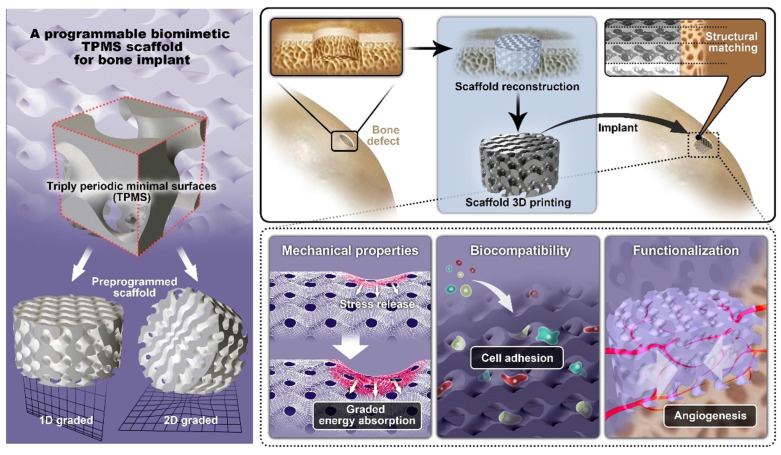
Biomimetic graded TPMS scaffold for bone implant. Reproduced from [12], licensed under CC BY 4.0.

One of the key reasons for the application of TPMS-based scaffolds in bone tissue engineering is their programmable mechanical properties, which can be controlled in the terms of elastic modulus, energy absorption, and stress distribution. For the improvement of the performance of bone implants, as shown in Figure 12, a graded TPMS scaffold with a controllable pore size was developed, which provides a subtle transition between cortical and trabecular bone regions. In comparison to uniform scaffolds, the graded scaffold design showed an elastic modulus value of 1158–4567 MPa, thereby facilitating better load distribution and minimizing stress concentrations [12]. Further, permeability studies have revealed that graded TPMS scaffolds facilitate better mass transport, a factor that is of paramount importance for the delivery of nutrients and cellular proliferation, therefore making them an extremely promising candidate for orthopedic applications.

To further enhance biomechanical compatibility, an orthotropic TPMS scaffold (AP and AG structures) was produced using Electron Beam Melting (EBM) and evaluated using compression tests and Computational Fluid Dynamics (CFD) simulations. Results showed that the elastic modulus of AG scaffolds was close to the modulus of cancellous bone, thereby making them an ideal option for load-bearing bone grafts [109]. Anisotropy of the scaffolds guarantees that mechanical properties are in harmony with natural bone stress distributions, thereby avoiding mechanical mismatch-induced implant failure.

In addition, fluid behavior has a massive impact on scaffold effectiveness, influencing the diffusion of nutrients, permeability, and wall shear stress (WSS). Studies of fluid dynamics in Diamond and Gyroid TPMS scaffolds using computational fluid dynamics (CFD) simulations found that Gyroid lattices are more permeable and possess lower pressure drops, making them most appropriate for the delivery of nutrients and enabling the tissue regeneration process [110].

**Figure 13:**
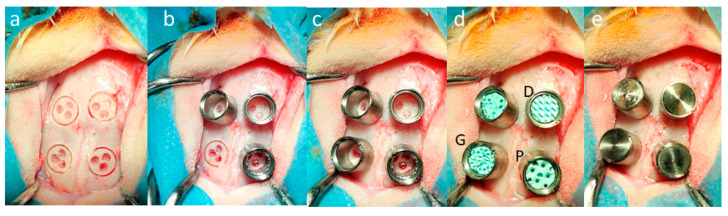
Calvarial bone augmentation model: (**a**) four circular sites (6.00 mm diameter) were prepared using a trephine, and bone ingrowth was initiated by creating three small perforations in the outer cortical plate; (**b**,**c**) titanium cylinders were inserted into the prepared slits; (**d**) TPMS scaffolds—D-diamond (D), G-gyroid (G), and P-primitive (P)—were placed within the cylinders; and (**e**) cylinders were sealed with press-fitted titanium lids. Reproduced from [111], licensed under CC BY 4.0.

A comparative in vivo and in vitro investigation also revealed that D-Diamond and G-Gyroid TPMS scaffolds are more osteoconductive and have better bone ingrowth than P-Primitive topologies. Titanium scaffolds manufactured through Selective Laser Melting (SLM) were implanted into rabbit femoral defects (Figure 13), whose micro-CT and histological examination revealed greater bone volume fraction and bone–implant contact for D and G structures. In vitro cell culture assays also substantiated the results, in which osteoblast growth and alkaline phosphatase (ALP) activity were significantly higher on D and G scaffolds, validating increased osteogenic differentiation. The results indicate that lattice morphology and curvature play a crucial role in cellular mechanotransduction and bone regeneration behavior; therefore, D and G TPMS are better designs for bone augmentation applications [111].

**Figure 14:**
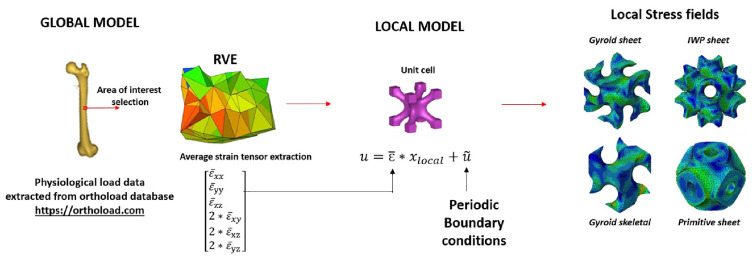
Illustration of the partial bone replacement approach. Reproduced from [113], licensed under CC BY 4.0.

With the evolution of AM techniques, functional grading of TPMS-based lattices is now more feasible, enabling tailored patient-specific implants. A numerical study was able to incorporate functional grading in TPMS scaffolds, with smooth gradation between various bone regions and porosity and mechanical performance optimization. They have introduced a complete numerical method for the design of TPMS-based lattices for the bone scaffold, considering cortical and trabecular bone needs [112]. Another study employed a multiscale numerical method to study four different TPMS unit cell topologies (Gyroid, Primitive, and IWP) in femoral bone implants. The results showed that Primitive sheet topology was the most suitable for patient-specific implants, as it provided the best balance between mechanical properties and physiological adaptation; Figure 14 shows how the numerical method has been used to study TPMS unit cell topologies for bone implants [113].

These findings are particularly relevant for orthopedic surgeons, as they can use statistical findings and R-factor analysis to select the most suitable TPMS topology for customized implants based on patient morphology and loading conditions. This approach significantly improves implant longevity and patient outcomes.

#### 6.3.2. Dental Applications

Apart from bone implant applications, TPMS scaffolds have also been used in dentistry, enhancing structural support as well as bio-compatibility. In one study, a bio-inspired hybrid TPMS structure was developed to optimize mechanical performance for dynamic loading conditions, thereby making it suitable for prostheses dentals [114]. In another study, TPMS-based scaffold designs were used to design zirconia-resin IPCs that optimized their tribological compatibility with natural teeth, reduced their wear, and improved their contact stress distribution. By combining TPMS zirconia scaffolds and resin, these IPCs are tribologically compatible with natural teeth and reduce wear and contact stress as well [115]. These results show the beneficial potential of TPMS structures in dental restoration as well as prosthetic devices.

**Figure 15:**
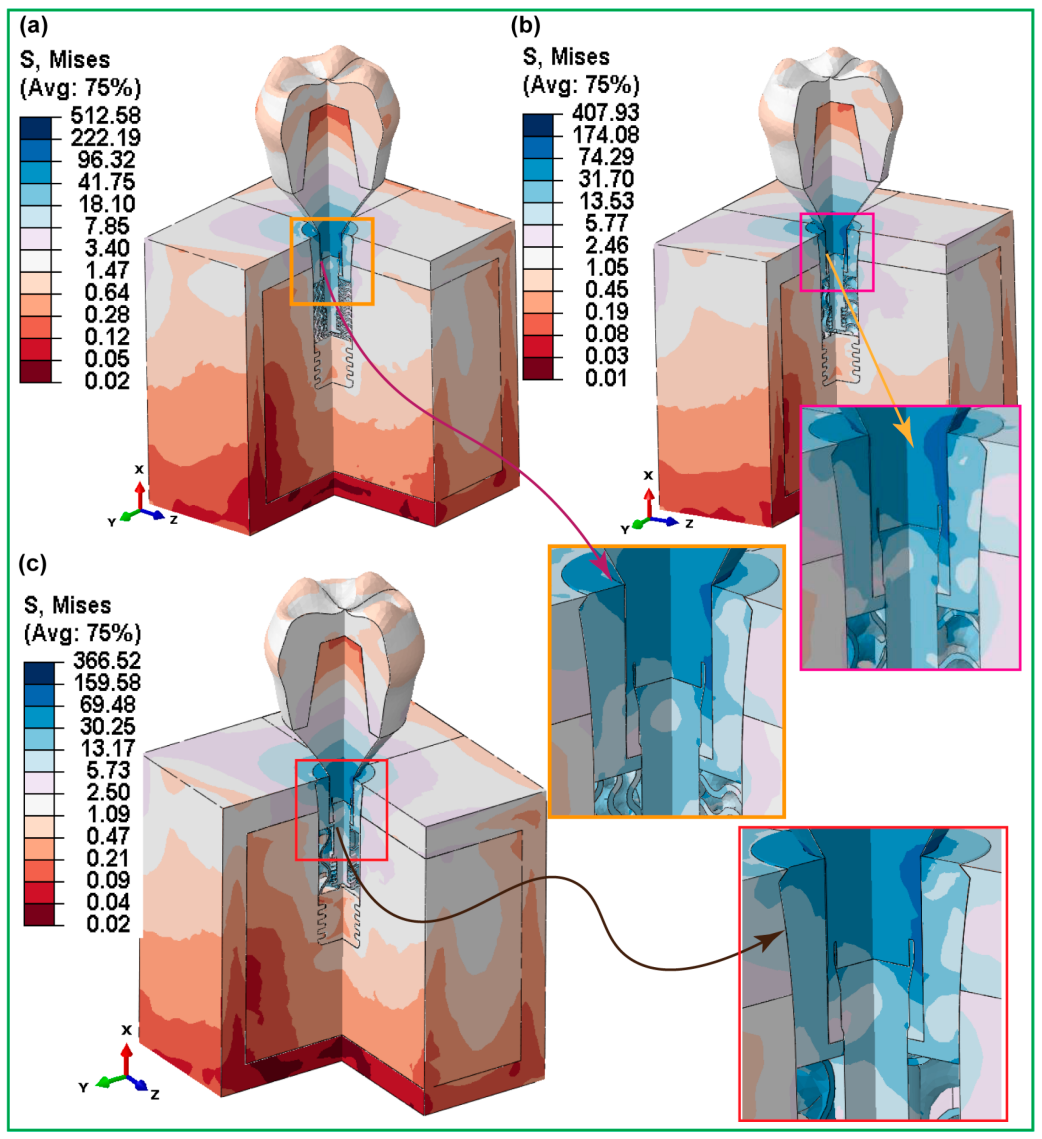
FEA stress contour plots illustrating the maximum von Mises stress distribution in hybrid latticed dental implants with varying cell sizes: (**a**) HI_111 (1 × 1 × 1), (**b**) HI_222 (2 × 2 × 2), and (**c**) HI_333 (3 × 3 × 3). The plots show both assembled views and detailed rectangular sections for comparison. Reproduced from [116], licensed under CC BY 4.0.

Traditional solid implants cause non-uniform stress distribution, which in turn reduces osseointegration and longevity. To overcome these issues, research focuses on hybrid gyroid-structured and fully gyroid-structured implants incorporating a solid neck using AM methods. Finite element analysis (FEA) indicates that hybrid implants, especially HI-222, have better stress distribution, reduced micromotions, and enhanced mechanical stiffness, leading to enhanced long-term performance (Figure 15) [116].

#### 6.3.3. Chondral Applications

**Figure 16:**
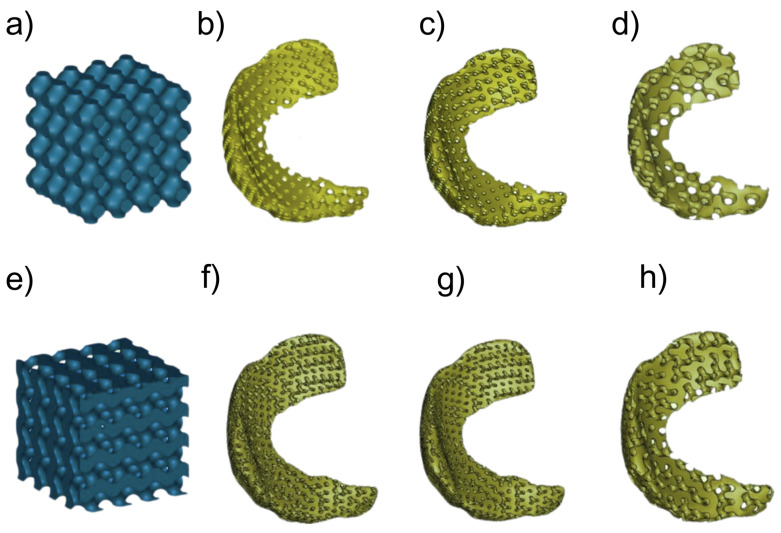
Meniscal TPMS scaffolds: (**a**) Schwarz P lattice, (**b**) P lattice implant with 47% porosity, (**c**) P lattice implant with 41% porosity, (**d**) P lattice implant with 47% porosity, (**e**) Gyroid lattice (**f**) G lattice implant with 45% porosity, (**g**) G lattice implant 37% porosity, (**h**) G lattice implant with 47% porosity Reproduced from [15], licensed under CC BY 4.0.

Apart from their orthopedic and dental uses, TPMS scaffolds have also shown promise in meniscal and cartilage tissue repair due to their stress-relieving ability in knee cartilage. In vitro, meniscal implants with primitive and gyroid TPMS surfaces, fabricated by Fused Deposition Modeling (FDM), significantly minimized compression and shear stresses, thus facilitating the maintenance of the natural semilunar shape of the cartilage. Of the various TPMS structures considered, the primitive TPMS with 41% porosity was found to exhibit better stress distribution and manufacturability, and thus is a potential candidate for clinical applications; meniscal TPMS scaffolds of primitive and gyroid surfaces are shown in Figure 16 [15].

**Figure 17:**
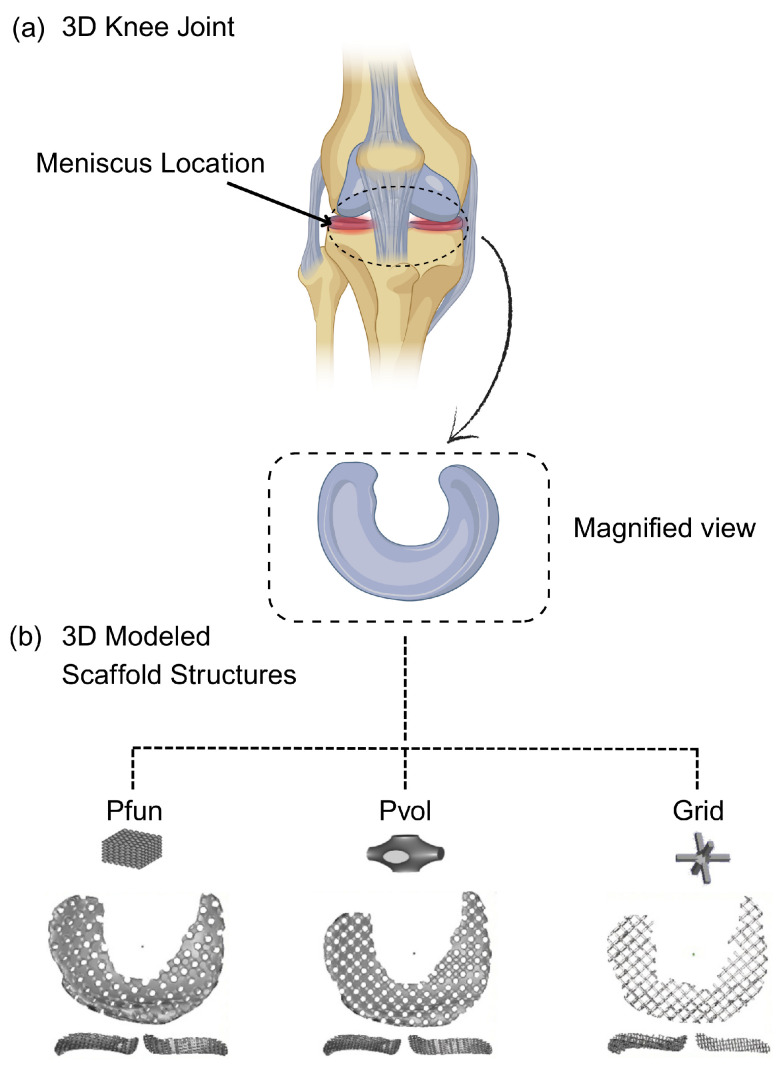
Meniscal scaffold. Reproduced from [117], licensed under CC BY 4.0.

Similarly, another group of researchers developed a meniscal scaffold using the TPMS method to restore cartilage protection in meniscal-injured patients. Meniscus injury is prevalent, and conventional treatments focus on pain relief but not function restoration, which can speed up osteoarthritis. This study has developed a scaffold that not only supports meniscus regeneration but also protects the underlying cartilage. 3D models of the knee joint were generated from CT and MRI images and the performance of the scaffold was tested using mechanical simulations; Figure 17 shows how meniscal scaffolds are being used. Results indicated that TPMS scaffolds outperformed conventional grid-based scaffolds in terms of mechanical strength and load transmission, proving their effectiveness in cartilage protection and meniscus regeneration [117].

As TPMS scaffolds continue to gain popularity in bone, dental, and cartilage regeneration, researchers are working to refine design and manufacturing criteria to enhance their mechanical, biological, and fluidic properties. The increasing popularity of TPMS-based biomimetic scaffolds is a testament to their pioneering contribution to biomedical engineering, leading the way to personalized, high-performance implants for a range of clinical uses. Future advances in AM, materials science, and computational modeling will continue to enhance the effectiveness and uptake of TPMS scaffolds in the clinic [118]. Table 4 enumerates the novel application of TPMS in biomimetic scaffolds.

## 7. Limitations and Practical Barriers

In spite of their encouraging potential, TPMS-based structures encounter several technical, clinical, and economic obstacles. Additive manufacturing also introduces intrinsic flaws in the form of surface roughness, pore-size deviation, and clogging of apertures that undermine permeability and mechanical precision. Polymer-based methods (SLA, DLP) have inherent issues with brittleness and shrinkage, while the metallic processes (SLM, EBM) cause several remnant stresses and necessitate high amounts of post-processing with accompanying cost increases. Exhibiting anisotropic, site-controllable mechanical properties remains a challenge due to poor reverse-design precision and disparities between predicted and produced geometries. From an application point of view, TPMS scaffolds exhibit inconsistency in osteoconductivity and bone formation in early stages based on pore geometry; sheet-type morphologies can suppress cellular ingrowth and regeneration of tissues. Clinically, a majority of TPMS implants remain limited to in vitro or in vivo studies in animals with no long-term in vivo proof under cyclic physiological loads. Issues such as sustaining long-term osseointegration under cyclic load, avoiding inflammatory/immunogenic response due to degradation of by-products, and maintaining consistent biocompatibility in variant TPMS geometries remain to be solved. Furthermore, the lack of universal clinical testing and regulatory guidelines still holds up clinical translation and widespread usage.

## 8. Discussion and Future Prospects

The absence of intuitive and robust design tools for integrating TPMS architectures into engineering applications has slowed their practical adoption. Software constraints often prevent users from tailoring complex TPMS topologies to specific functional objectives, despite well-documented advantages such as improved energy absorption, enhanced mechanical efficiency, and reduced weight. As a result, relatively few studies have focused on applying TPMS structures to context-driven, performance-sensitive designs. To advance the field, future research should focus on developing multi-scale finite element analysis frameworks capable of capturing the mechanical response of TPMS lattices across different resolution levels. Establishing standardized design-of-experiment protocols would also support the generation of statistically meaningful data for comparative and optimization studies. Equally important is the creation of accessible design platforms that allow researchers and engineers to seamlessly integrate TPMS lattices into customized applications.

TPMS-based scaffolds have been shown to be very promising in biomedical applications, but there are some key areas that require further research to maximize their effectiveness. One such key area is the creation of enhanced functional grading approaches, which provide seamless integration among various zones of the scaffold to accurately mimic the heterogeneous bone structure. Furthermore, multi-material TPMS scaffolds with bioactive metals, polymers, and ceramics would add strength and biological compatibility to provide long-term implant stability. Another key area is real-time biomechanical modeling and AI-aided scaffold design, where machine learning can predict the the amount of stress each patient will impart to the scaffold and assist in optimizing the scaffold designs. Moreover, research on bioresorbable TPMS scaffolds could result in implants that erode slowly as new tissue replaces them, which would reduce the need for secondary surgeries. Lastly, live and long-term studies on these scaffolds are required to fully understand how they function biologically and to create optimized TPMS scaffolds for more use in clinics. Advances in these areas will significantly enhance the reliability, applicability, and long-term success of TPMS-based implants for regenerative medicine.

## 9. Conclusions

In this review, the recent research on design, properties, optimization, and application of TPMS structures were summarized. To meet the geometric requirements, TPMS porous structures should be designed to closely resemble natural architectures, allowing them to inherit exceptional functional advantages such as graded porosity, heterogeneous characteristics, and multiscale pores. To meet performance requirements, the multifunctional performance of TPMS can be effectively tailored through the design flexibility offered by various geometric design approaches. TO has significantly advanced the design of TPMS structures by enabling precise control over mechanical properties, graded porosity, and structural efficiency. MSTO further enhances TPMS by concurrently optimizing macro- and microstructural features, leading to functionally graded and seamlessly connected architectures. Recent developments, such as variable-density gyroid geometries and multi-lattice topology frameworks, have improved mechanical performance and manufacturability. However, challenges remain in simultaneously optimizing global and local topologies while maintaining computational efficiency. Future research should focus on refining optimization methods and integrating data-driven approaches to further enhance TPMS design for advanced engineering applications.

TPMS structures significantly enhance thermal management and composite performance owing to their high surface-to-volume ratio and adjustable porosity. In heat sinks, they lower thermal resistance and improve cooling efficiency, while in interpenetrating phase composites (IPCs), they enable lightweight, high-strength materials with superior stress distribution and energy absorption. In biomimetic applications, TPMS scaffolds have shown high potential in orthopedic, dental, and cartilage applications owing to high permeability and interconnected porosity and bone-like mechanical compliance. Graded TPMS scaffolds with an elastic modulus in the range 1158–4567 MPa provide smooth load transmission along with superior nutrient transport, whereas Gyroid and Diamond morphologies maximize cell proliferation and permeability. Fischer–Koch S and cancellous bone/IWP morphologies are useful for cancellous bone and femoral stem applications, due to optimal strength and physiological acceptability. In dental applications, wear resistance and stress behavior are improved with TPMS-based zirconia–resin IPCs, and maximum osseointegration is achieved with gyroid hybrid implants. In cartilage applications, shear stress and joint geometry are preserved with primitive TPMS scaffolds, and high potentials in meniscus regeneration and patient-specific implants are achieved. In summary, TPMS-based biomimetic scaffolds have high potential in personalized cartilage, dental, and bone regeneration, and can yield second-generation patient-specific implants.

AM plays a key role in the fabrication of highly complex, high-fidelity TPMS structures required in biomimetic tissue engineering. High geometric fidelity scaffolds are fabricated with optimized native-like mechanical properties in the processes of AM. SLA and DLP are key for polymeric/ceramic TPMS, yielding high precision and smooth surfaces; SLA, for instance, limits unit cell deviations to less than 1.1%. In the case of metallic TPMS, SLM enables support-free printing of Gyroids, with the resultant mechanical properties ideally matching trabecular bone. However, the minimum pore size and optimal grade of surfaces are a key area of concern, with the SLM surface roughness typically in the range of 3.2–4.4 µm, which impacts the tissue ingrowth and degradation of the scaffold. In spite of remarkable progress, the clinical translation of TPMS scaffolds is hindered by some root technological limitations and key challenges. Current AM techniques have some manufacturing defects such as aperture plugging, extra surface roughing, and thickness deviations in the walls, making experimental characteristics like strength and permeability deviate substantially from ideal numerical projections. In addition, precise reproduction of native bone anisotropy for personalized implants via reverse design is extremely difficult. The main challenge is translational maturity; TPMS structures are limited to preclinical studies. Systematic validation needs to be conducted for the purpose of establishing long-term stability and consistent mechanical behavior with realistic cyclic loadings prior to human implantation.

## Figures and Tables

**Table 1:** Summary of AM techniques for TPMS.

Manufacturing Technique	Key Findings/ Achievements	Common Defects/ Limitations	Best Application Area	Achieved Scale/Resolution	Availability/Cost
SLA (Stereolithography)	High precision and smooth surface finish; ideal for polymer TPMS scaffolds.	Brittle resin, shrinkage, limited build size.	Bone scaffolds, tissue engineering—smooth surface enhances cell adhesion and biological response.	Layer thickness: 25–50 µm	Widely available; moderate cost
DLP (Digital Light Processing)	High-resolution ceramic/polymer TPMS with tunable porosity and strength up to 937 MPa.	Light scattering, sintering shrinkage, small build area.	Biomedical scaffolds, thermal ceramics—precise control for bioactive materials.	Grain size: 1–8 µm	Commercial; medium cost
FDM (Fused Deposition Modeling)	Cost-effective fabrication; tunable porosity via infill patterns.	Poor surface finish, anisotropy, limited resolution.	Structural prototypes, polymer scaffolds—low-cost for functional TPMS models.	Pore size: 650–1000 µm; layer height: 0.12 mm	Highly available; very low cost
SLS (Selective Laser Sintering)	Complex polymer/metal TPMS with good accuracy and strength.	Surface roughness, incomplete sintering, powder waste.	Aerospace lattice cores—strong, lightweight designs with complex geometries.	Layer thickness: 50–80 µm	Commercial; high cost
SLM (Selective Laser Melting)	Fully dense metallic TPMS; adjustable porosity for strength/stiffness control.	Residual stress, high roughness, post-processing required.	Load-bearing implants, metallic lattices—excellent mechanical and biocompatibility performance.	Layer thickness: 30 µm	Commercial industrial; very high cost
EBM (Electron Beam Melting)	Produces high-strength metal TPMS with lower residual stresses.	High surface roughness, limited resolution.	Orthopedic and aerospace parts—dense, stress-free metallic lattices.	Layer thickness: 50 µm	Industrial; very high cost
2PP (Two-Photon Polymerization)	Ultra-high precision sub-micron TPMS fabrication.	Small build volume, slow, expensive.	Micro-tissue scaffolds, photonics—ideal for cellular-level control.	0.2 µm voxel size	Lab-scale; extremely high cost
Robocasting (Direct Ink Writing)	Bioceramic TPMS with gradient porosity and bioactivity.	Cracking during drying/sintering, low mechanical strength.	Bone tissue engineering—tunable degradation and pore structure.	Nozzle Ø 0.41 mm	Lab to pilot scale; low–medium cost
Micro-SLA	Fine polymeric TPMS scaffolds with high surface quality and accuracy.	Shrinkage, polymerization defects, low mechanical load capacity.	Soft-tissue scaffolds—precise, smooth features for biocompatibility.	Layer thickness: 5 µm	Laboratory-scale; moderate cost

**Table 2:** Recent research on application of TPMSs in heat sinks.

Sr#	Author(s)	Structure Type	Primary Application	Analysis Method/ Manufacturing Process	Comparison/ Benchmark	Methodology & Validation	Key Findings	Limitations or Future Works	Source DOI
1	Mian et al., 2025	Schwarz-P	Electronics heat sink cooling	CFD on AM materials, no physical fabrication	Compared with traditional plate-fin heat sink; validated via pin-fin model	Simulated flow and thermal performance using SST k-ω model; varied unit cell sizes (5–20 mm) and porosity (65–75%); validation done	Smaller cell sizes improve heat transfer but increase pressure drop; 5 mm Schwarz-P is 50% more efficient; lower porosity improves resistance	AM limits: surface defects, high computation; needs experimental validation	https://doi.org/10.1016/j.csite.2025.106273
2	Ansari & Duwig 2024	Gyroid	Microprocessor cooling	CFD on AM materials, no physical fabrication	Compared Gyroid (GHS) vs. pin-fin (PHS) under porosity 0.5–0.8	Conjugate heat transfer; analyzed flow, temperature, pressure via streamlines; hotspot analysis	GHS outperformed PHS by 30% lower resistance; 3D helical flow enhanced transfer; lower porosity, higher flow rate improved performance	Limited porosity range; high pressure drop; explore graded porosity, roughness, TPMS orientation	https://doi.org/10.1016/j.enconman.2024.118918
3	Modrek et al., 2022	Gyroid	Thermal management in AM heat sinks	Modeled in nTopology + Abaqus thermal simulation	Compared SIMP and homogenized gyroid (solid/sheet) designs	Topology optimization (SIMP & homogenized); FE homogenization; thermal conductivity simulations	Gradient-mapped gyroid-sheet had highest conductivity, lowest temperature; outperformed SIMP/solid	Experimental validation pending; only steady-state conduction studied; explore other TPMS (D/P)	https://doi.org/10.1016/j.csite.2022.102161
4	Baobaid et al., 2022	Diamond, Gyroid	Passive natural convection cooling	CFD (Star-CCM+); AlSi10Mg properties	Compared with pin-fin and metal foam heat sinks; orientation studied	Simulations under enclosures; radiation included; derived Nu–Ra correlation	Gyroid-sheet 50% better than pin-fin; radiation 17–23% of heat dissipation; horizontal 11.5% better; outperformed Al foam by 44%	Future work: porosity effects with varied TPMS geometries	https://doi.org/10.1016/j.csite.2022.101944
5	Saghir & Rahman 2024	Gyroid	Forced convection cooling (water)	COMSOL simulation + experiments vs. Al 6061-T6 foam	Compared TPMS vs. metal foam; parallel vs. perpendicular flow	FEM (Navier–Stokes, conduction); varied porosities, flow directions	TPMS superior to foam; gyroid gave uniform cooling; parallel flow more effective; optimal PEC at ɸ = 0.8	Impingement jets less effective; test 3D-printed TPMS; study turbulent regimes	https://doi.org/10.3390/fluids9120297
6	Chen et al., 2025	Gyroid	Thermal dissipation	SLM (AlSi10Mg, 33 µm avg.)	Compared TPMS vs. plate-fin heat sink	CFD (COMSOL) + experiments (1–10 L/min, 500 W load)	TPMS had 60% higher efficiency, lower pressure drop/pump power; improved temperature uniformity	Focused on Gyroid only; future work: other TPMS, thinner fins, turbulent flow	https://doi.org/10.1016/j.tsep.2025.103499
7	Wang et al., 2024	Gyroid	Electronics cooling	3D-printed TPMS samples	Compared 3 TPMS geometries + baseline	Simulations + experiments under cycling; temperature and HTC analyzed	TPMS outperformed conventional; better uniformity; 12.9–16.6% HTC improvement	Limited to one PCM type/power; future work on PCM enhancement, variable heat loads	https://doi.org/10.1016/j.ijheatmasstransfer.2024.126078

**Table 3:** Recent research on application of TPMS in IPCs.

Sr#	Author(s)	Structure Type	Primary Application	Manufacturing Process	Comparison/ Benchmark	Methodology & Validation	Key Findings	Limitations or Future Works	Source DOI
1	Song et al., 2023	Diamond, IWP, Gyroid	Mechanical energy absorption, impact protection	PolyJet-based multi-material 3D printing (VB+ as reinforcement, PP as matrix)	Compared IPCs with single-phase matrix/reinforcement; validated via FEM	Experimental compression testing + FEM (RVE and full model) validation; IPC strength and SEA compared with sum of constituents; studied effect of equivalent density and deformation modes	IPCs showed up to 497% strength increase over constituent phases; 33% SEA improvement; Diamond best SEA (24.6 J/g), Gyroid highest strength (84 MPa); strong synergistic effect	FEM assumes perfect bonding; dynamic/turbulent loads not explored; future work could explore more material combinations and real-world loads	https://doi.org/10.1016/j.tws.2023.111210
2	Singh & Karathanasopoulos 2024	Gyroid, IWP, P-Cell TPMS and stochastic spinodal	Mechanical and dynamic damping performance of architected IPCs	Additive manufacturing with ceramic-epoxy co-continuous phases; with and without whisker reinforcement	Compared SEA and damping performance against advanced architected materials (steel TPMS, Ti TPMS, TPU, CNT foams, PA) using Ashby plots	Static & dynamic tests (SEA, stress-strain, DMA 25–70 Hz); FEA & DIC for stress and crack analysis	IPCs show 10–30× strength over ceramic alone (peak > 140 MPa); SEA up to 18.5 J/g; damping matches wood/bone; spinodal better at 20%, TPMS better at 30% content	Dynamic tests limited to 70 Hz; only 20–30% phase content studied; no biomedical validation; needs calibrated dynamic IPC models	https://doi.org/10.1016/j.compscitech.2024.110632
3	Xie et al., 2024	Primitive, IWP	Bone scaffolds—improved strength, toughness, energy absorption	Photopolymerization-based additive manufacturing and PUF foaming	Compared IPCs with constituent scaffolds (PMMA, PUF); benchmarked with trabecular bone (2.96–4.01 MPa, 4–430 MPa modulus); validated simulation	Compression tests; SEM, CT, DIC, FEA; analyzed stress distribution, failure, and crack propagation	I-WP IPCs improved strength (134%), toughness (73%), and energy absorption (236%); within human trabecular bone range; better stress uniformity and synergy than Primitive	Tensile/fatigue testing missing; reduced PUF at high density affects performance; further study needed for crack propagation and bone integration	https://doi.org/10.1016/j.compstruct.2024.118526
4	Santos et al., 2024	Gyroid, Diamond, Primitive; Aluminium–Alumina	Mechanical performance improvement; energy absorption	DLP-based 3D printing of alumina TPMS + aluminium infiltration via investment casting	Compared different TPMS geometries and volume fractions; evaluated against cast aluminium	Compression testing (ISO 13314); SEM; Micro-CT; XRD; densification & shrinkage measurements	Diamond and Gyroid IPCs showed 10% higher plateau stress and 6.8% higher SEA vs. Primitive; IPCs improved compressive offset stress by 6% over Al alloy; pseudo-ductile failure in ceramics	Fatigue/tensile testing missing; Al–Al_2_O_3_ interface issues; further study needed on bonding and alternative materials	https://link.springer.com/article/10.1007/s40964-024-00698-7
5	Guo et al., 2023	Primitive	Structural composites with enhanced compressive strength and energy absorption	Metal AM via Micro-SLM (SS316L); epoxy (EPOLAM 2040) injection molding to form IPCs	Compared empty P-lattices and IPCs; modified vs. original P-lattice; FEM validation; benchmarked against linear sum of epoxy + lattice	Quasi-static compression testing; SEM imaging; FEM via Abaqus; evaluated internal energy of lattice, epoxy, and IPC	Modified TPMS (SP & BP) enhanced SEA (up to 49.6 J/g) and strength; improved crushing resistance; FEM showed 136% ↑ in lattice energy and 21% ↑ in epoxy contribution	Limited to static compression; not tested for fatigue; fracture/delamination observed; mechanical behavior depends on lattice direction; explore multi-axial loading	https://doi.org/10.1016/j.compositesb.2022.110351

**Table 4:** Recent research on application of TPMS in biomimetic scaffolds.

Sr#	Author(s)	Structure Type	Primary Application	Manufacturing Process	Comparison/ Benchmark	Methodology & Validation	Key Findings	Limitations or Future Works	Source DOI
1	Raffaele Pugliese et al., 2024	Primitive, Gyroid	Knee meniscal implants	FDM printing proposed	Compared to healthy knee	FEA for stress analysis	TPMS-based meniscal implants prevent higher magnitude compression and shear stress on articular cartilage	Effects of variations in pore size, porosity, and pore shape of the meniscal implant should be studied	https://doi.org/10.1016/j.slast.2023.04.004
2	Rati Verma et al., 2022	Primitive, Gyroid	Segmental bone defect (Femur)	Designed for AM using Ti6Al4V, not fabricated	Compared with solid scaffold	FEA for porosity-dependent stress & modulus; biomechanical model of femur used	P scaffold improved stress transfer by 76% vs. solid; stiffness reduced from 107 GPa (solid) to 4.2–29.6 GPa matching bone; stretching-dominant deformation	Need experimental validation; suggested experimental permeability analysis and clinical validation	https://doi.org/10.3390/coatings12060839
3	Hou et al., 2022	Graded Primitive	Dental implants	SLM (CP-Ti; Porosities: 48–68%)	Compared P50–P70 vs. human bone	FEA simulation, compression testing, permeability testing, in vitro biocompatibility, PCR analysis for osteogenic genes	P60 scaffold showed optimal mechanical properties (E = 9.7 GPa, σy = 163 MPa); permeability within bone range; strong osteogenic differentiation	Slight porosity deviation, surface roughness reduced permeability, clinical validation needed	https://doi.org/10.1016/j.bioadv.2022.213018
4	Dabaja et al., 2025	Gyroid, Voronoi Stochastic Lattice	Dental implants	SLM with Ti6Al4V ELI Grade 23	Compared 3 porous architectures vs. dense implant	MicroCT, SEM, confocal microscopy, RNA extraction, CFD for permeability	TPMS solid gyroid showed highest resolution, controlled porosity (220 µm), 4× higher RNA, best cell adhesion, uniform distribution, highest permeability	In vitro only; SLM-induced partially melted particles; future CFD needed with real blood conditions	https://link.springer.com/article/10.1186/s40729-025-00618-6
5	Jiaqi Ma et al., 2024	Primitive, Gyroid, I-WP, Diamond, Fischer–Koch S	Bone regeneration (dental, cortical, trabecular, breast, ocular)	3D printing (primarily FDM); hydrogel infusion for soft tissue	Compared TPMS scaffolds with conventional implants and natural bone	Literature review of clinical & experimental findings; mechanical behavior, permeability, antibacterial use, anisotropic behavior	TPMS scaffolds offer high permeability, bone-mimicking structure, favorable mechanical properties (20–60 MPa), enhanced osseointegration	Limited to 3D printing; deviation between designed and printed geometry; rough surface; small-aperture clogging; only preclinical studies	https://doi.org/10.1177/20417314241263689
6	Shen et al., 2023	Diamond, Gyroid, IWP, Diamond, Gyroid	Femoral bone defect regeneration in rabbits	DLP-based 3D printing of Ca_0.94_Mg_0.06_SiO_3_ scaffolds (>50% porosity, >500 µm)	Compared 5 TPMS geometries (3 non-sheet vs. 2 sheet-type)	In vivo rabbit model, µCT, histology, mechanical testing, BV/TV, Tb.N, Tb.Th, BS/TS	Diamond and Gyroid scaffolds showed highest bone ingrowth; sheet-type better compressive strength but lower osteoconductivity	Early-stage bone formation inhibited in sheet-type pores; osteoconductivity strongly depends on pore geometry; further studies on remodeling needed	https://doi.org/10.1016/j.bioactmat.2023.02.012

## Data Availability

No new data were created or analyzed in this study. Data sharing is not applicable to this article.

## Nomenclature

*L*	Unit cell size of TPMS
*W*	Spatial frequency or wavenumber, given by $W=\frac{2\pi }{L}$
ϕ	Level-set approximation
*c*	Threshold value or level-set constant which defines the relative density of TPMS
α, β, γ	Parameters controlling cell size and shape
*G*	Spatial coordinate function defining the shape of the transition between different regions
*k*	Parameter controlling the width of the transition region
$K_{\omega }$	Stiffness matrix of the domain ω, dependent on material distribution
$u_{\omega }$	Displacement field in domain ω, dependent on material distribution
P(x)	External load vector applied at position *x*
χ(x)	Material indicator function: 1 if x∈ω, 0 if x∈Ω∖ω
ω	Design domain where material is present
Ω	Total domain including both material and void regions
